# Supplementary material for: Sociodemographic factors, biomarkers and comorbidities associated with post-acute COVID-19 sequelae in UK Biobank
Source: Nat Commun. 2025 Jul 30;16:7009. doi: 10.1038/s41467-025-62354-0 (PMC12311030; doi:10.1038/s41467-025-62354-0)
Supplement: Supplementary file 1 — Supplementary Information [file 41467_2025_62354_MOESM1_ESM.pdf]

Supplementary materials

Supplementary Note 1. Exploratory data analysis, data curation and correlation analysis results.

Oestradiol and Rheumatoid Factor were found to have significant missing data (85% and 92%, respectively) and were therefore not included in further analyses (see Supplementary Table 8). The proportion of outliers and imputed values for the remaining variables are reported in Supplementary Table 9. The covariate with highest imputed data was lipoprotein (a) (25.25%), and the one with highest number of outliers was glucose (0.92%). Descriptive analyses of all covariates after data curation can be found in Supplementary Table 10.

Apolipoprotein B and cholesterol were found to be highly correlated with LDL Direct levels (r = 0.96 and r = 0.95, respectively). We excluded both variables and only explored LDL levels. Apolipoprotein A was found to be highly associated with HDL Cholesterol (r = 0.92), and only the latter one was then included in the analysis (see Supplementary Figure 2). Overall, we excluded 11 biomarkers and 2 comorbidities (Supplementary Table 11) due to their strong association with another biomarker or comorbidity.

Supplementary Note 2. Pre-existing symptoms.

The Health and well-being online questionnaire assessed the length of each symptom by asking people for how long they have been suffering the specific condition (Supplementary Table 5). The answer could be either (1) less than two weeks, (2) two to four weeks, (3) four to twelve weeks, (4) more than twelve weeks, (5) do not know, or (6) prefer not to answer. If participants reported options (1), (2), (5), (6), we assumed that the patient did not have pre-existing symptoms. If participants reported option (3) we assumed that the symptoms started four weeks before answering the questionnaire (28 days). If participants reported option (4), we directly assumed that the participants had pre-existing symptoms, as the date when the symptoms started could not be determined. Afterwards, we excluded those participants who reported option (4) or those whose date of answering the questionnaire minus the number of days they have been suffering the symptoms was before the date of the infection.

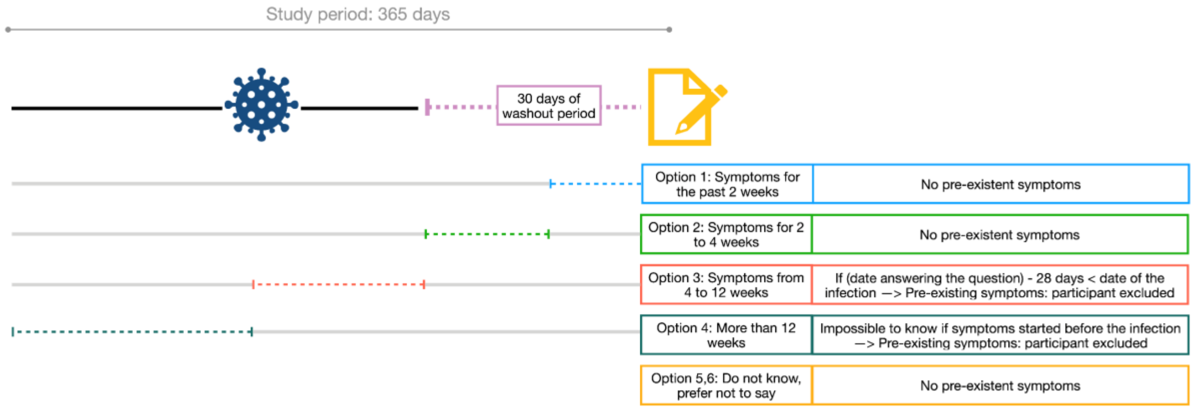

For those WHO symptoms that were mapped to more than one UK Biobank symptom, the duration was determined by the symptom with longest duration.

Supplementary Note 3. Data curation and correlation analysis details.

We used multiple imputation with chained equations to handle missing data. Five different datasets were generated with all the observed covariates included in the imputation model. We defined values greater than the mean plus three times the standard deviation of a given variable as outliers. These outliers were then capped at this threshold value.

To ensure comparability across biomarkers, we standardised their scales to have a mean of zero and a standard deviation of one using z-scores. Moreover, the ethnic background variable was further transformed to a binary variable (White/Non-white).

We calculated pairwise correlation coefficients for variables within the three categories: sociodemographic, biomarker, or comorbidity. For any pair of variables with a strong correlation ($|r| > 0.5$), we retained only one based on two criteria: (1) fewer imputed values, and (2) knowledge of clinical relevance.

Supplementary Note 4. Categorisation of variables.

Biomarkers

Biomarkers showing a potential non-linear association with the study outcome were divided using the 4 quantiles.

Body mass index

Average weight included participants with a weight less than 25kg/m^2.

Overweight included participants with weight >= 25kg/m^2 and 30kg/m^2.

Obesity included participants with weight >= 30kg/m^2.

Reference group is average weight. Underweight group was not considered due to insufficient sample size.

Index of multiple deprivation (IMD)

Values were divided across 4 quantiles.

Very affluent group included participants with an IMD smaller than the value of the first quantile.

Affluent group included participants with IMD >= q1 and < q2.

Average group included participants with IMD >= q2 and < q3.

Deprived group included participants with IMD >= q3 and < q4.

Very deprived group included participants with IMD >= q4.

Reference group is very affluent.

Age when infected

Participants divided across those aged <55: >=55 and <60; >= 60 and <65; >=65 and < 70; >= 70 and < 75; and >= 75 when infected by COVID-19.

Supplementary Table 1. LASSO coefficients of the selected variants associated with Long COVID.

Lasso features	Lasso coefficients
Baseline characteristics, optimal λ = 0.0001	
Age when infected	-0.0040
Sex	-0.1523
Body mass index	0.0371
Ethnic background	0.1265
Index of multiple deprivation	0.0095
Smoking status	0.0853
Biomarkers, optimal λ = 0.0001	
Age when infected	-0.0115
Sex	-0.2763
Alanine aminotransferase	0.0002
Alkaline phosphatase	-0.0153
C reactive protein - Q1	0.1830
C reactive protein - Q2	0.4091
C reactive protein - Q3	0.1884
C reactive protein - Q4	0.7677
C reactive protein - Q5	0.2376
Calcium	0.0092
Cystatin C	0.0486
HbA1c	0.0640
HDL-cholesterol - Q1	-0.4361
HDL-cholesterol - Q2	-0.3779
HDL-cholesterol - Q3	-0.3759
HDL-cholesterol - Q4	-0.6508
IGF-1	-0.0748
LDL direct	0.0049
Lipoprotein (a)	0.0445
Phosphate	0.0066
SHBG	-0.0378
Total bilirubin	0.0344
Triglycerides	0.0882
Urate	0.0406
Urea	-0.0272
Vitamin D	0.0379
Comorbidities, optimal λ = 0.000570164272280748	
Age when infected	-0.0061
Sex	-0.0952
Cancer - metastatic	-0.2892
Cerebrovascular disease	-0.0745
Chronic obstructive pulmonary disease (COPD)	0.2623
Chronic kidney disease	0.3430
Diabetes	0.1509
Fracture	-0.0766
Liver disease – moderate to severe	0.0198
Peptic ulcer	0.1643
Rheumatoid arthritis	0.1677

Supplementary Table 2. LASSO coefficients of the selected variants associated with PACS.

Lasso features	Lasso coefficients
Baseline characteristics, optimal λ = 0.0001	
Age when infected	0.0685
Sex	0.3828
Body mass index	0.0643
Ethnic background	0.1601
Index of multiple deprivation	0.0125
Smoking status	0.2158
Biomarkers, optimal λ = 0.0001	
Age when infected	0.0491
Sex	0.3480
Alkaline phosphatase - Q3	0.2012
Alkaline phosphatase - Q5	0.0691
C reactive protein - Q1	-0.1178
C reactive protein - Q2	0.0515
C reactive protein - Q3	0.1879
C reactive protein - Q5	0.4343
Calcium - Q2	-0.0421
Cystatin C	0.2241
HbA1c - Q1	-0.1322
HbA1c - Q3	0.3915
HbA1c - Q5	0.6338
HDL-cholesterol - Q2	-0.0106
HDL-cholesterol - Q3	-0.0253
HDL-cholesterol - Q5	-0.0143
IGF-1 - Q2	-0.1801
IGF-1 - Q3	-0.0783
IGF-1 - Q4	0.0171
LDL direct - Q1	-0.1508
LDL direct - Q2	-0.2308
LDL direct - Q3	-0.4099
LDL direct - Q4	0.0506
Lipoprotein (a)	0.0568
Phosphate	0.0289
SHBG	-0.0246
Triglyceride - Q5	0.0887
Urate - Q5	0.1613
Urea - Q2	-0.0417
Urea - Q3	-0.0653
Vitamin D	-0.0004
Comorbidities, optimal λ = 0.0001	
Age when infected	0.0447
Sex	0.3156
Cancer	0.1689
Cancer - metastatic	0.2874
Cerebrovascular disease	0.1306
Chronic kidney disease	0.4210
Congestive heart failure	0.9241
COPD	0.4246
Dementia	0.0082
Diabetes	0.5907
Fracture	0.0549
Hemiplegia	0.0673
Liver disease - moderate to severe	0.0605
Myocardial infarction	0.8888
Peptic ulcer	0.3815
Peripheral vascular disease	0.3640
Rheumatoid arthritis	0.4076

Supplementary Table 3. Variance inflator factor (VIF) of the Long COVID adjusted analysis.

**Risk factor**	**GVIF**
Age when infected	1.48
Body mass index	1.58
Ethnic background	1.05
Index of multiple deprivation	1.06
Sex	2.20
Smoking status	1.08
Cancer - metastatic	1.01
Cerebrovascular disease	1.02
Chronic obstructive pulmonary disease (COPD)	1.02
Chronic kidney disease	1.07
Diabetes	1.23
Fracture	1.02
Liver disease - moderate to severe	1.02
Peptic ulcer	1.04
Rheumatoid arthritis	1.02
Alanine aminotransferase	1.39
Alkaline phosphatase	1.21
C reactive protein	1.40
Calcium	1.19
Cystatin C	1.54
HbA1c	1.36
HDL-cholesterol	1.82
IGF-1	1.21
LDL direct	1.27
Lipoprotein (a)	1.02
Phosphate	1.14
SHBG	1.84
Total bilirubin	1.16
Triglycerides	1.72
Urate	2.18
Urea	1.23
Vitamin D	1.12

Supplementary Table 4. Variance inflation factor (VIF) of the PACS adjusted analysis.

Risk factor	GVIF (after)
Ethnic background	1.10
Body mass index	1.57
Index of multiple deprivation	1.09
Age when infected	1.49
Sex	1.77
Smoking status	1.16
Cancer	1.15
Cancer - metastatic	1.08
Cerebrovascular disease	1.28
Congestive heart failure	1.18
Chronic kidney disease	1.29
COPD	1.10
Dementia	1.07
Diabetes	1.51
Fracture	1.04
Hemiplegia	1.20
Liver disease - moderate to severe	1.03
Myocardial infarction	1.19
Peptic ulcer	1.09
Peripheral vascular disease	1.09
Rheumatoid arthritis	1.06
Cystatin C	1.64
Lipoprotein (a)	1.02
Phosphate	1.14
SHBG	1.63
Vitamin D	1.16
Alkaline phosphatase	1.15
C-reactive protein	1.43
Calcium	1.15
HbA1c	1.48
HDL-Cholesterol	1.79
IGF-1	1.21
LDL direct	1.31
Triglyceride	1.66
Urate	1.77
Urea	1.26

Supplementary Table 5. Example of questions asked by the UK Biobank health and well-being online questionnaire for each one of the symptoms.

**Questions asked about symptom X.**	**Possible answers**
Currently suffering from X.	1. Yes.<br>2. No.<br>3. Do not know.<br>4. Prefer not to answer.
Length of time suffering from X.	1. Less than two weeks.<br>2. Two to four weeks.<br>3. Four to twelve weeks.<br>4. More than twelve weeks.<br>5. Do not know.<br>6. Prefer not to answer.
Extent affected by X.	1. It is not limiting me.<br>2. It is causing me to avoid, reduce or spread out my usual activities.<br>3. Do not know.<br>4. Prefer not to answer.

Supplementary Table 6. UK Biobank symptoms mapped to the World Health Organisation (WHO) Delphi consensus.

WHO symptom	UK Biobank symptom
Abdominal pain	Abdominal pain / tummy ache
Menstrual and period problems	-
Altered smell/taste	Loss or change in sense of smell<br>Loss or change in sense of taste
Anxiety	Problems relating to mood anxiety and emotions
Blurred vision	Vision problems
Chest pain	Chest pain<br>Pain on breathing
Cognitive dysfunction / brain fog	Problems communicating<br>Problems thinking
Cough	Persistent cough
Depression	-
Dizziness	Dizziness / light headedness
Fatigue	Mild fatigue<br>Severe fatigue
Intermitted fever	Fever
Gastrointestinal issues	Gastrointestinal issues
Headache	Headache
Memory issues	-
Joint pain	Joint pain or swelling of joint
Muscle pain / spasms	Muscle pain / achy muscles
Neuralgias	-
New onset allergies	New allergy or intolerance
Pins and needles sensations	Numbness or tigling somewhere in the body
Post-exertional malaise	Post-exertional symptom exacerbation
Shortness of breath	Shortness of breath or trouble breathing
Sleep disorders	Difficulty sleeping<br>Unrestful sleep
Tachycardia / palpitations	Postural tachycardia
Tinnitus and other hearing issues	Tinnitus<br>Hearing loss<br>Hearing issues

Supplementary Table 7. Post-acute COVID-19 sequelae diagnoses used to phenotype the condition.

Organ system	Sequela	ICD10 code
Cardiovascular	Acute coronary disease	I24, I240, I241, I248, I249
Cardiovascular	Angina	I20, I200, I201, I208, I209
Cardiovascular	Atrial fibrillation	I480, I481, I482
Cardiovascular	Atrial flutter	I483, I484
Cardiovascular	Bradycardia	R001
Cardiovascular	Cardiac arrest	I46, I460, I461, I469
Cardiovascular	Cariogenic shock	R570
Cardiovascular	Heart failure	I50, I500, I501, I509
Cardiovascular	Ischemic cardiomyopathy	I255
Cardiovascular	Myocardial infarction	I21, I210, I211, I212, I213, I214, I219 I21X, I22, I220, I221, I228, I229
Cardiovascular	Myocarditis	I514
Cardiovascular	Non ischemic cardiomyopathy	I42, I420, I421, I422, I423, I424, I425, I426, I427, I428, I429, I43, I430, I431, I432, I438 B332
Cardiovascular	Pericarditis	I30, I300, I301, I308, I309, I311, I312, I313, I318, I319, I32, I320, I321, I328
Cardiovascular	Tachycardia	R000
Cardiovascular	Ventricular arrhythmias	I490, I470, I471, I472
Coagulation	Anaemia	D60, D600, D601, D608, D609, D61, D610, D611, D612, D613, D618, D619, D63, D630, D631, D638, D64, D640, D641, D642, D643, D644, D648, D649
Coagulation	Coagulation defect	D689
Coagulation	Deep vein thrombosis	I801, I802, I803, I81, I81X
Coagulation	Pulmonary embolism	I26, I260, I269
Coagulation	Venous thrombotic embolism	I82, I820, I822, I823, I828, I829

Supplementary Table 8. Exploratory data analysis before data curation.

Risk factor	UK Biobank dataset	Distribution
**N**	**502,180**	
Baseline characteristics		
**Sex (%)**		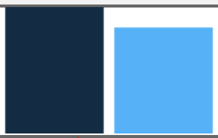
Female	273,185 (54.4)	
Male	228,995 (45.6)	
Counts	502,180 (100)	
Missings	0 (0)	
**Year of birth**		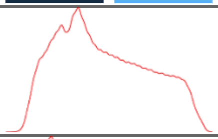
Mean (SD)	1,951.54 (8.12)	
Quantiles (0.05, 0.25, 0.5, 0.75, 0.95)	1,940, 1,945, 1,950, 1,958, 1,966	
Counts	502,180 (100)	
Missings	0 (0)	
**BMI (kg/m2)**		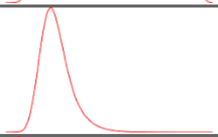
Mean (SD)	27.43 (4.8)	
Quantiles (0.05, 0.25, 0.5, 0.75, 0.95)	20, 24, 26, 29, 36	
Counts	499,075 (99.38)	
Missings	3,105 (0.62)	
**Index of multiple deprivation**		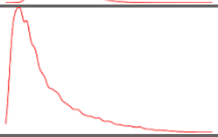
Mean (SD)	17.34 (14.09)	
Quantiles (0.05, 0.25, 0.5, 0.75, 0.95)	3, 7, 12, 23, 47	
Counts [N (%)]	489,454 (97.47)	
Missings	12,726 (2.53)	
**Ethnic background (%)**		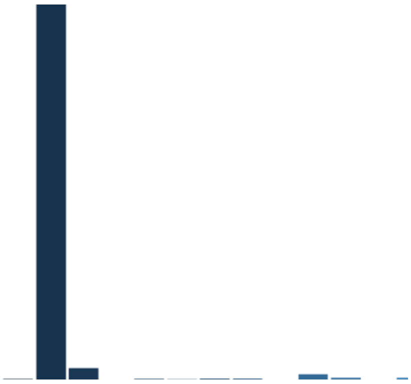
White	0 (0)	
British	442,335 (88.08)	
Irish	13,194 (2.63)	
Any other white background	16,309 (3.25)	
Mixed	49 (0.01)	
White and Black Caribbean	619 (0.12)	
White and Black African	425 (0.08)	
White and Asian	830 (0.17)	
Any other mixed background	1,028 (0.2)	
Asian or Asian British	43 (0.01)	
Indian	5,946 (1.18)	
Pakistani	1,833 (0.37)	
Bangladeshi	236 (0.05)	
Any other Asian background	1,814 (0.36)	
Black or Black British	27 (0.01)	
Caribbean	4,510 (0.9)	
African	3,390 (0.68)	
Any other Black background	122 (0.02)	
Chinese	1,573 (0.31)	
Other ethnic group	4,553 (0.91)	
Counts	499,404 (99.45)	
Missings	2,776 (0.55)	
**Smoking status (%)**		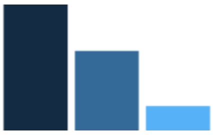
Never	273,351 (54.43)	
Previous	172,940 (34.44)	
Current	52,940 (10.54)	
Counts	499,231 (99.41)	
Missings	2,949 (0.59)	
Biomarkers		
**Alanine aminotransferase (U/L)**		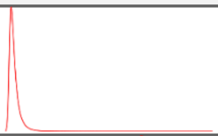
Mean (SD)	23.55 (14.18)	
Q05, Q25, Q50, Q75, Q95	10.76, 15.41, 20.13, 27.40, 47.03	
Counts	469,101 (93.41)	
Missings	33,079 (6.59)	
**Albumin (g/L)**		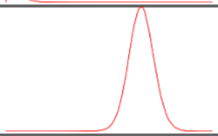
Mean (SD)	45.21 (2.63)	
Q05, Q25, Q50, Q75, Q95	40, 43, 45, 46, 49	
Counts	429,800 (85.59)	
Missings	72,380 (14.41)	
**Alkaline Phosphatase (U/L)**		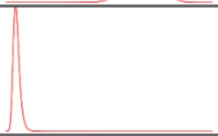
Mean (SD)	83.67 (26.46)	
Q05, Q25, Q50, Q75, Q95	51, 67, 80, 95, 125	
Counts	469,302 (93.45)	
Missings	32,878 (6.55)	
**Apolipoprotein A (g/L)**		
Mean (SD)	1.54 (0.27)	

Risk factor	UK Biobank dataset	Distribution
Q05, Q25, Q50, Q75, Q95	1, 1, 1, 1, 2	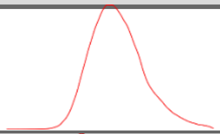
Counts	427,235 (85.08)	
Missings	74,945 (14.92)	
**Apolipoprotein B (g/L)**		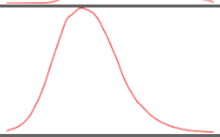
Mean (SD)	1.03 (0.24)	
Q05, Q25, Q50, Q75, Q95	0, 0, 1, 1, 1	
Counts	466,909 (92.98)	
Missings	35,271 (7.02)	
**Aspartate aminotransferase (U/L)**		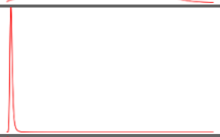
Mean (SD)	26.23 (10.66)	
Q05, Q25, Q50, Q75, Q95	17, 21, 24, 28, 40	
Counts	467,497 (93.09)	
Missings	34,683 (6.91)	
**C-reactive protein (mg/L)**		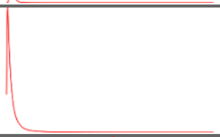
Mean (SD)	2.6 (4.36)	
Q05, Q25, Q50, Q75, Q95	0, 0, 1, 2, 8	
Counts	468,268 (93.25)	
Missings	33,912 (6.75)	
**Calcium (mmol/L)**		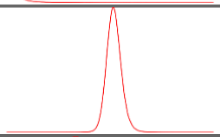
Mean (SD)	2.38 (0.09)	
Q05, Q25, Q50, Q75, Q95	2, 2, 2, 2, 2	
Counts	429,656 (85.56)	
Missings	72,524 (14.44)	
**Cholesterol (mmol/L)**		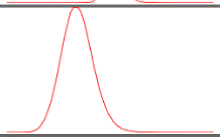
Mean (SD)	5.69 (1.14)	
Q05, Q25, Q50, Q75, Q95	3, 4, 5, 6, 7	
Counts	469,289 (93.45)	
Missings	32,891 (6.55)	
**Creatinine (umol/L)**		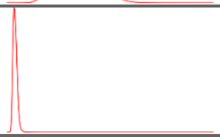
Mean (SD)	72.31 (18.55)	
Q05, Q25, Q50, Q75, Q95	51, 61, 70, 80, 97	
Counts	469,057 (93.4)	
Missings	33,123 (6.6)	
**Cystatin C (mg/L)**		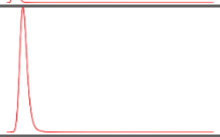
Mean (SD)	0.91 (0.18)	
Q05, Q25, Q50, Q75, Q95	0, 0, 0, 0, 1	
Counts	469,258 (93.44)	
Missings	32,922 (6.56)	
**Direct bilirubin (umol/L)**		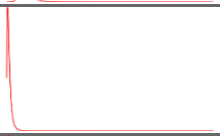
Mean (SD)	1.83 (0.85)	
Q05, Q25, Q50, Q75, Q95	1, 1, 1, 2, 3	
Counts	398,358 (79.33)	
Missings	103,822 (20.67)	
**Gamma glutamyltransferase (U/L)**		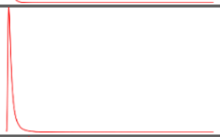
Mean (SD)	37.39 (42.09)	
Q05, Q25, Q50, Q75, Q95	12, 18, 26, 41, 94	
Counts	469,042 (93.4)	
Missings	33,138 (6.6)	
**Glucose (mmol/L)**		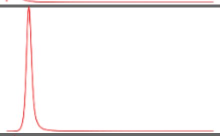
Mean (SD)	5.13 (1.24)	
Q05, Q25, Q50, Q75, Q95	4, 4, 4, 5, 6	
Counts	429,291 (85.49)	
Missings	72,889 (14.51)	
**HbA1c (mmol/mol)**		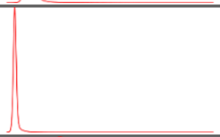
Mean (SD)	36.13 (6.78)	
Q05, Q25, Q50, Q75, Q95	29, 32, 35, 37, 45	
Counts	466,209 (92.84)	
Missings	35,971 (7.16)	
**HDL-Cholesterol (mmol/L)**		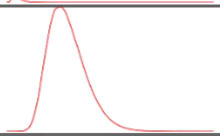
Mean (SD)	1.45 (0.38)	
Q05, Q25, Q50, Q75, Q95	0, 1, 1, 1, 2	
Counts	429,595 (85.55)	
Missings	72,585 (14.45)	
**IGF-1 (nmol/L)**		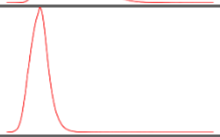
Mean (SD)	21.4 (5.7)	
Q05, Q25, Q50, Q75, Q95	12, 17, 21, 24, 30	
Counts	466,743 (92.94)	
Missings	35,437 (7.06)	
**LDL direct (mmol/L)**		

Risk factor	UK Biobank dataset	Distribution
Mean (SD)	3.56 (0.87)	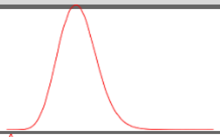
Q05, Q25, Q50, Q75, Q95	2, 2, 3, 4, 5	
Counts	468,407 (93.27)	
Missings	33,773 (6.73)	
**Lipoprotein (a) (nmol/L)**		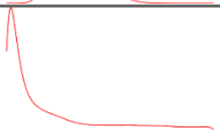
Mean (SD)	44.64 (49.21)	
Q05, Q25, Q50, Q75, Q95	4, 9, 21, 61, 158	
Counts	375,386 (74.75)	
Missings	126,794 (25.25)	
**Oestradiol (pmol/L)**		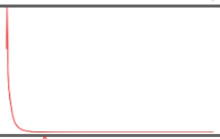
Mean (SD)	461.06 (431.04)	
Q05, Q25, Q50, Q75, Q95	182, 216, 315, 536, 1,219	
Counts	76,597 (15.25)	
Missings	425,583 (84.75)	
**Phosphate (mmol/L)**		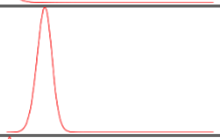
Mean (SD)	1.16 (0.16)	
Q05, Q25, Q50, Q75, Q95	0, 1, 1, 1, 1	
Counts	428,975 (85.42)	
Missings	73,205 (14.58)	
**Rheumatoid factor (IU/ml)**		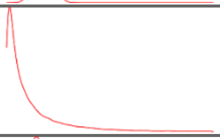
Mean (SD)	24.56 (19.86)	
Q05, Q25, Q50, Q75, Q95	10, 12, 16, 27, 69	
Counts	41,289 (8.22)	
Missings	460,891 (91.78)	
**SHBG (nmol/L)**		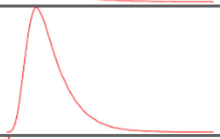
Mean (SD)	51.63 (27.78)	
Q05, Q25, Q50, Q75, Q95	19, 32, 45, 63, 104	
Counts	425,571 (84.74)	
Missings	76,609 (15.26)	
**Testosterone (nmol/L)**		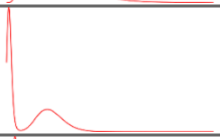
Mean (SD)	6.56 (6.05)	
Q05, Q25, Q50, Q75, Q95	0, 1, 3, 11, 16	
Counts	424,926 (84.62)	
Missings	77,254 (15.38)	
**Total bilirubin (umol/L)**		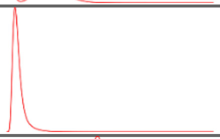
Mean (SD)	9.13 (4.43)	
Q05, Q25, Q50, Q75, Q95	4, 6, 8, 10, 17	
Counts	467,261 (93.05)	
Missings	34,919 (6.95)	
**Total protein (g/L)**		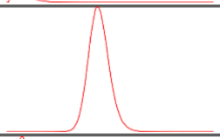
Mean (SD)	72.51 (4.12)	
Q05, Q25, Q50, Q75, Q95	66, 69, 72, 75, 79	
Counts	429,329 (85.49)	
Missings	72,851 (14.51)	
**Triglycerides (mmol/L)**		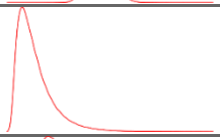
Mean (SD)	1.75 (1.03)	
Q05, Q25, Q50, Q75, Q95	0, 1, 1, 2, 3	
Counts	468,914 (93.38)	
Missings	33,266 (6.62)	
**Urate (umol/L)**		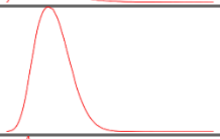
Mean (SD)	309.22 (80.44)	
Q05, Q25, Q50, Q75, Q95	189, 250, 303, 360, 450	
Counts	468,725 (93.34)	
Missings	33,455 (6.66)	
**Urea (mmol/L)**		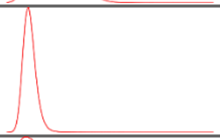
Mean (SD)	5.4 (1.4)	
Q05, Q25, Q50, Q75, Q95	3, 4, 5, 6, 7	
Counts	468,971 (93.39)	
Missings	33,209 (6.61)	
**Vitamin D (nmol/L)**		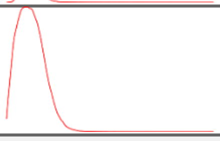
Mean (SD)	48.61 (21.11)	
Q05, Q25, Q50, Q75, Q95	18, 32, 46, 62, 85	
Counts	448,065 (89.22)	
Missings	54,115 (10.78)	
Comorbidities		
**Acquired immunodeficiency syndrome (AIDS) (%)**		
Cases	180 (0.04)	
Controls	502,000 (99.96)	
Counts	502,180 (100)	

Risk factor		UK Biobank dataset	Distribution
Missings		0 (0)	
**Asthma (%)**			
	Cases	49,840 (9.92)	
	Controls	452,340 (90.08)	
Counts		502,180 (100)	
Missings		0 (0)	
**Cancer (%)**			
	Cases	165,983 (33.05)	
	Controls	336,197 (66.95)	
Counts		502,180 (100)	
Missings		0 (0)	
**Cancer - metastatic (%)**			
	Cases	26,281 (5.23)	
	Controls	475,899 (94.77)	
Counts		502,180 (100)	
Missings		0 (0)	
**Cerebrovascular disease (%)**			
	Cases	30,142 (6)	
	Controls	472,038 (94)	
Counts		502,180 (100)	
Missings		0 (0)	
**Congestive heart failure (%)**			
	Cases	20,375 (4.06)	
	Controls	481,805 (95.94)	
Counts		502,180 (100)	
Missings		0 (0)	
**Chronic obstructive pulmonary disease (COPD) (%)**			
	Cases	68,001 (13.54)	
	Controls	434,179 (86.46)	
Counts		502,180 (100)	
Missings		0 (0)	
**Chronic kidney disease (%)**			
	Cases	48,065 (9.57)	
	Controls	454,115 (90.43)	
Counts		502,180 (100)	
Missings		0 (0)	
**Dementia (%)**			
	Cases	6,695 (1.33)	
	Controls	495,485 (98.67)	
Counts		502,180 (100)	
Missings		0 (0)	
**Diabetes (%)**			
	Cases	46,820 (9.32)	
	Controls	455,360 (90.68)	
Counts		502,180 (100)	
Missings		0 (0)	
**Diabetes - organ damage (%)**			
	Cases	7,619 (1.52)	
	Controls	494,561 (98.48)	
Counts		502,180 (100)	
Missings		0 (0)	
**Fracture (%)**			
	Cases	45,620 (9.08)	
	Controls	456,560 (90.92)	
Counts		502,180 (100)	
Missings		0 (0)	
**Hemiplegia (%)**			
	Cases	5,448 (1.08)	
	Controls	496,732 (98.92)	
Counts		502,180 (100)	
Missings		0 (0)	
**Liver disease - mild (%)**			
	Cases	17,838 (3.55)	
	Controls	484,342 (96.45)	
Counts		502,180 (100)	
Missings		0 (0)	
**Liver disease - moderate to severe (%)**			

Risk factor		UK Biobank dataset	Distribution
	Cases	9,745 (1.94)	
	Controls	492,435 (98.06)	
Counts		502,180 (100)	
Missings		0 (0)	
**Myocardial infarction (%)**			
	Cases	26,778 (5.33)	
	Controls	475,402 (94.67)	
Counts		502,180 (100)	
Missings		0 (0)	
**Peptic ulcer (%)**			
	Cases	56,085 (11.17)	
	Controls	446,095 (88.83)	
Counts		502,180 (100)	
Missings		0 (0)	
**Peripheral vascular disease (%)**			
	Cases	14,597 (2.91)	
	Controls	487,583 (97.09)	
Counts		502,180 (100)	
Missings		0 (0)	
**Rheumatoid arthritis (%)**			
	Cases	16,870 (3.36)	
	Controls	485,310 (96.64)	
Counts		502,180 (100)	
Missings		0 (0)	

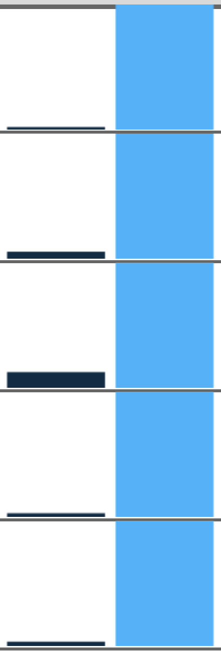

Supplementary Table 9. Number of missing data and outliers for each one of the variables.

Risk factors	Number of outliers (%)	Number of imputed values (%)
Baseline characteristics		
Sex	0 (0)	0 (0)
Year of birth	0 (0)	0 (0)
Ethnic background	0 (0)	899 (0.18)
Body mass index	4,119 (0.82)	3,105 (0.62)
Smoking status	0 (0)	2,949 (0.59)
Index of multiple deprivation	654 (0.13)	12,726 (2.53)
Biomarkers		
Alanine aminostransferase	3,991 (0.79)	33,079 (6.59)
Albumin	292 (0.06)	72,380 (14.41)
Alkaline phosphatase	1,227 (0.24)	32,878 (6.55)
Apolipoprotein A	136 (0.03)	74,945 (14.92)
Apolipoprotein B	244 (0.05)	35,271 (7.02)
Aspartate aminotransferase	1,049 (0.21)	34,683 (6.91)
C reactive protein	2,879 (0.57)	33,912 (6.75)
Calcium	340 (0.07)	72,524 (14.44)
Cholesterol	1,004 (0.2)	32,891 (6.55)
Creatinine	921 (0.18)	33,123 (6.6)
Cystatin C	1,131 (0.23)	32,922 (6.56)
Direct bilirubin	599 (0.12)	103,822 (20.67)
Gamma glutamyltransferase	2,668 (0.53)	33,138 (6.6)
Glucose	4,598 (0.92)	72,889 (14.51)
HbA1c	636 (0.13)	35,971 (7.16)
HDL-cholesterol	766 (0.15)	72,585 (14.45)
IGF-1	2,311 (0.46)	35,437 (7.06)
LDL direct	863 (0.17)	33,773 (6.73)
Lipoprotein (a)	0 (0)	126,794 (25.25)
Phosphate	209 (0.04)	73,205 (14.58)
SHBG	4,222 (0.84)	76,609 (15.26)
Testosterone	1,037 (0.21)	77,254 (15.38)
Total bilirubin	2,018 (0.4)	34,919 (6.95)
Total protein	696 (0.14)	72,851 (14.51)
Triglycerides	3,026 (0.6)	33,266 (6.62)
Urate	938 (0.19)	33,455 (6.66)
Urea	837 (0.17)	33,209 (6.61)
Vitamin D	104 (0.02)	54,115 (10.78)
Comorbidities		
AIDS	0 (0)	0 (0)
Asthma	0 (0)	0 (0)
Cancer	0 (0)	0 (0)
Cancer - metastatic	0 (0)	0 (0)
Cerebrovascular disease	0 (0)	0 (0)
Congestive heart failure	0 (0)	0 (0)
Chronic kidney disease	0 (0)	0 (0)
COPD	0 (0)	0 (0)
Dementia	0 (0)	0 (0)
Diabetes	0 (0)	0 (0)
Diabetes – organ damage	0 (0)	0 (0)
Fracture	0 (0)	0 (0)
Hemiplegia	0 (0)	0 (0)
Liver disease - mild	0 (0)	0 (0)
Liver disease – moderate to severe	0 (0)	0 (0)
Myocardial infarction	0 (0)	0 (0)
Peptic ulcer	0 (0)	0 (0)
Peripheral vascular disease	0 (0)	0 (0)
Rheumatoid arthritis	0 (0)	0 (0)

Supplementary Table 10. Exploratory data analysis after data curation.

Risk factor	UK Biobank dataset	Distribution
**N**	**502,180**	
Baseline characteristics		
**Sex (%)**		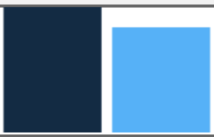
Female	273,185 (54.4)	
Male	228,995 (45.6)	
Counts	502,180 (100)	
Missings	0 (0)	
**Year of birth**		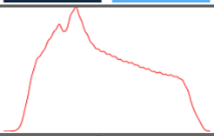
Mean (SD)	1,951.54 (8.12)	
Quantiles (0.05, 0.25, 0.5, 0.75, 0.95)	1,940, 1,945, 1,950, 1,958, 1,966	
Counts	502,180 (100)	
Missings	0 (0)	
**BMI (kg/m2)**		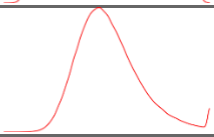
Mean (SD)	27.39 (4.63)	
Quantiles (0.05, 0.25, 0.5, 0.75, 0.95)	20, 24, 26, 29, 36	
Counts	502,180 (100)	
Missings	0 (0)	
**Index of multiple deprivation**		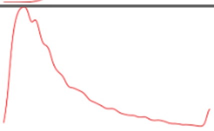
Mean (SD)	17.24 (13.7)	
Quantiles (0.05, 0.25, 0.5, 0.75, 0.95)	3, 7, 12, 23, 47	
Counts [N (%)]	502,180 (100)	
Missings	0 (0)	
**Ethnic background (%)**		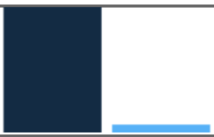
White	473,202 (94.23)	
Non-white	28,978 (5.77)	
Counts	502,180 (100)	
Missings	0 (0)	
**Smoking status (%)**		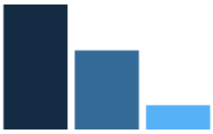
Never	274,814 (54.72)	
Previous	173,985 (34.65)	
Current	53,381 (10.63)	
Counts	502,180 (100)	
Missings	0 (0)	
Biomarkers		
**Alanine aminostransferase**		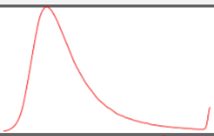
Mean (SD)	0 (1)	
Q05, Q25, Q50, Q75, Q95	-1.08, -0.67, -0.26, 0.37, 2.08	
Counts	502,180 (100)	
Missings	0 (0)	
**Albumin**		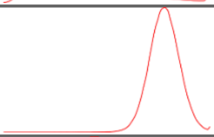
Mean (SD)	0 (1)	
Q05, Q25, Q50, Q75, Q95	-1.61, -0.66, -0.01, 0.65, 1.65	
Counts	502,180 (100)	
Missings	0 (0)	
**Alkaline phosphatase**		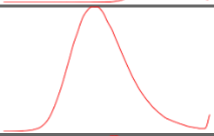
Mean (SD)	0 (1)	
Q05, Q25, Q50, Q75, Q95	-1.39, -0.70, -0.13, 0.55, 1.84	
Counts	502,180 (100)	
Missings	0 (0)	
**Apolipoprotein A**		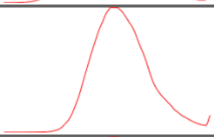
Mean (SD)	0 (1)	
Q05, Q25, Q50, Q75, Q95	-1.44, -0.71, -0.10, 0.58, 1.88	
Counts	502,180 (100)	
Missings	0 (0)	
**Apolipoprotein B**		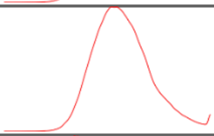
Mean (SD)	0 (1)	
Q05, Q25, Q50, Q75, Q95	-1.53, -0.71, -0.06, 0.63, 1.78	
Counts	502,180 (100)	
Missings	0 (0)	
**Aspartate aminotransferase**		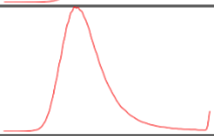
Mean (SD)	0 (1)	
Q05, Q25, Q50, Q75, Q95	-1.16, -0.65, -0.20, 0.39, 1.87	
Counts	502,180 (100)	
Missings	0 (0)	
**C-reactive Protein**		
Mean (SD)	0 (1)	
Q05, Q25, Q50, Q75, Q95	-0.71, -0.58, -0.35, 0.12, 2.14	
Counts	502,180 (100)	

Risk factor	UK Biobank dataset	Distribution
Missings	0 (0)	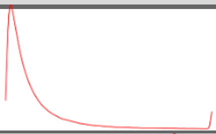
**Calcium**		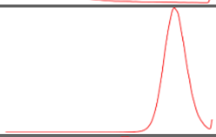
Mean (SD)	0 (1)	
Q05, Q25, Q50, Q75, Q95	-1.54, -0.66, -0.04, 0.61, 1.71	
Counts	502,180 (100)	
Missings	0 (0)	
**Cholesterol**		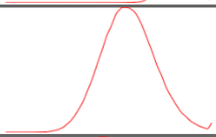
Mean (SD)	0 (1)	
Q05, Q25, Q50, Q75, Q95	-1.58, -0.69, -0.04, 0.65, 1.72	
Counts	502,180 (100)	
Missings	0 (0)	
**Creatinine**		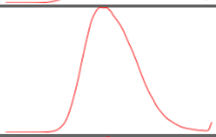
Mean (SD)	0 (1)	
Q05, Q25, Q50, Q75, Q95	-1.41, -0.72, -0.12, 0.60, 1.76	
Counts	502,180 (100)	
Missings	0 (0)	
**Cystatin C**		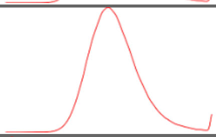
Mean (SD)	0 (1)	
Q05, Q25, Q50, Q75, Q95	-1.39, -0.68, -0.12, 0.53, 1.81	
Counts	502,180 (100)	
Missings	0 (0)	
**Direct bilirubin**		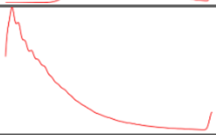
Mean (SD)	0 (1)	
Q05, Q25, Q50, Q75, Q95	-1.00, -0.72, -0.29, 0.38, 2.16	
Counts	502,180 (100)	
Missings	0 (0)	
**Gamma glutamyltransferase**		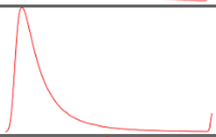
Mean (SD)	0 (1)	
Q05, Q25, Q50, Q75, Q95	-0.80, -0.60, -0.33, 0.18, 2.07	
Counts	502,180 (100)	
Missings	0 (0)	
**Glucose**		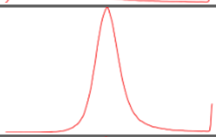
Mean (SD)	0 (1)	
Q05, Q25, Q50, Q75, Q95	-1.15, -0.54, -0.16, 0.28, 1.77	
Counts	502,180 (100)	
Missings	0 (0)	
**HbA1c**		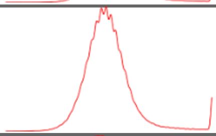
Mean (SD)	0 (1)	
Q05, Q25, Q50, Q75, Q95	-1.28, -0.58, -0.13, 0.38, 1.75	
Counts	502,180 (100)	
Missings	0 (0)	
**HDL-cholesterol**		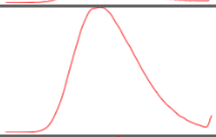
Mean (SD)	0 (1)	
Q05, Q25, Q50, Q75, Q95	-1.41, -0.73, -0.13, 0.60, 1.87	
Counts	502,180 (100)	
Missings	0 (0)	
**IGF-1**		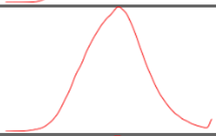
Mean (SD)	0 (1)	
Q05, Q25, Q50, Q75, Q95	-1.57, -0.70, -0.02, 0.62, 1.72	
Counts	502,180 (100)	
Missings	0 (0)	
**LDL Direct**		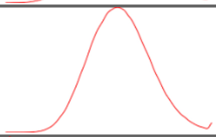
Mean (SD)	0 (1)	
Q05, Q25, Q50, Q75, Q95	-1.57, -0.71, -0.05, 0.65, 1.73	
Counts	502,180 (100)	
Missings	0 (0)	
**Lipoprotein (a)**		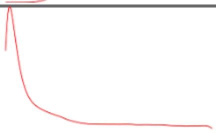
Mean (SD)	0 (1)	
Q05, Q25, Q50, Q75, Q95	-0.81, -0.71, -0.48, 0.35, 2.31	
Counts	502,180 (100)	
Missings	0 (0)	
**Phosphate**		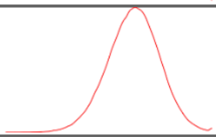
Mean (SD)	0 (1)	
Q05, Q25, Q50, Q75, Q95	-1.68, -0.66, 0.02, 0.68, 1.61	
Counts	502,180 (100)	
Missings	0 (0)	
**SHBG**		

Risk factor	UK Biobank dataset	Distribution
Mean (SD)	0 (1)	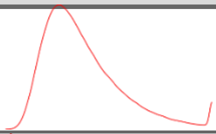
Q05, Q25, Q50, Q75, Q95	-1.21, -0.72, -0.23, 0.49, 2.05	
Counts	502,180 (100)	
Missings	0 (0)	
**Testosterone**		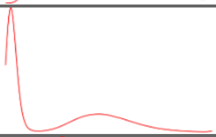
Mean (SD)	0 (1)	
Q05, Q25, Q50, Q75, Q95	-0.93, -0.86, -0.71, 0.86, 1.75	
Counts	502,180 (100)	
Missings	0 (0)	
**Total bilirubin**		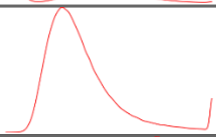
Mean (SD)	0 (1)	
Q05, Q25, Q50, Q75, Q95	-1.11, -0.67, -0.25, 0.37, 2.16	
Counts	502,180 (100)	
Missings	0 (0)	
**Total protein**		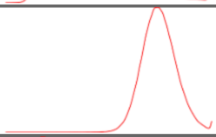
Mean (SD)	0 (1)	
Q05, Q25, Q50, Q75, Q95	-1.55, -0.68, -0.05, 0.63, 1.73	
Counts	502,180 (100)	
Missings	0 (0)	
**Triglycerides**		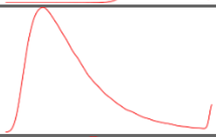
Mean (SD)	0 (1)	
Q05, Q25, Q50, Q75, Q95	-1.12, -0.73, -0.26, 0.45, 2.12	
Counts	502,180 (100)	
Missings	0 (0)	
**Urate**		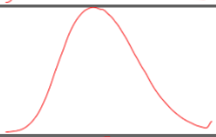
Mean (SD)	0 (1)	
Q05, Q25, Q50, Q75, Q95	-1.49, -0.73, -0.08, 0.65, 1.78	
Counts	502,180 (100)	
Missings	0 (0)	
**Urea**		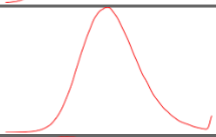
Mean (SD)	0 (1)	
Q05, Q25, Q50, Q75, Q95	-1.46, -0.69, -0.10, 0.58, 1.81	
Counts	502,180 (100)	
Missings	0 (0)	
**Vitamin D**		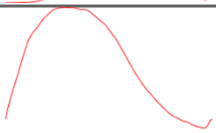
Mean (SD)	0 (1)	
Q05, Q25, Q50, Q75, Q95	-1.45, -0.78, -0.08, 0.67, 1.78	
Counts	502,180 (100)	
Missings	0 (0)	
Comorbidities		
**Acquired immunodeficiency syndrome (AIDS) (%)**		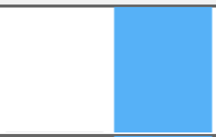
Cases	180 (0.04)	
Controls	502,000 (99.96)	
Counts	502,180 (100)	
Missings	0 (0)	
**Asthma (%)**		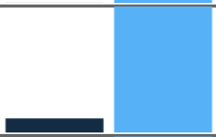
Cases	49,840 (9.92)	
Controls	452,340 (90.08)	
Counts	502,180 (100)	
Missings	0 (0)	
**Cancer (%)**		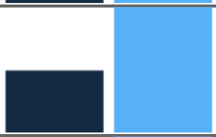
Cases	165,983 (33.05)	
Controls	336,197 (66.95)	
Counts	502,180 (100)	
Missings	0 (0)	
**Cancer - metastatic (%)**		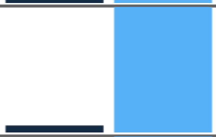
Cases	26,281 (5.23)	
Controls	475,899 (94.77)	
Counts	502,180 (100)	
Missings	0 (0)	
**Cerebrovascular disease (%)**		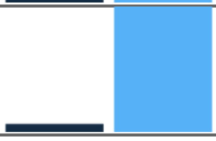
Cases	30,142 (6)	
Controls	472,038 (94)	
Counts	502,180 (100)	
Missings	0 (0)	
**Congestive heart failure (%)**		
Cases	20,375 (4.06)	
Controls	481,805 (95.94)	
Counts	502,180 (100)	

Risk factor	UK Biobank dataset	Distribution
Missings	0 (0)	
**Chronic obstructive pulmonary disease (COPD) (%)**		
Cases	68,001 (13.54)	
Controls	434,179 (86.46)	
Counts	502,180 (100)	
Missings	0 (0)	
**Chronic kidney disease (%)**		
Cases	48,065 (9.57)	
Controls	454,115 (90.43)	
Counts	502,180 (100)	
Missings	0 (0)	
**Dementia (%)**		
Cases	6,695 (1.33)	
Controls	495,485 (98.67)	
Counts	502,180 (100)	
Missings	0 (0)	
**Diabetes (%)**		
Cases	46,820 (9.32)	
Controls	455,360 (90.68)	
Counts	502,180 (100)	
Missings	0 (0)	
**Diabetes - organ damage (%)**		
Cases	7,619 (1.52)	
Controls	494,561 (98.48)	
Counts	502,180 (100)	
Missings	0 (0)	
**Fracture (%)**		
Cases	45,620 (9.08)	
Controls	456,560 (90.92)	
Counts	502,180 (100)	
Missings	0 (0)	
**Hemiplegia (%)**		
Cases	5,448 (1.08)	
Controls	496,732 (98.92)	
Counts	502,180 (100)	
Missings	0 (0)	
**Liver disease - mild (%)**		
Cases	17,838 (3.55)	
Controls	484,342 (96.45)	
Counts	502,180 (100)	
Missings	0 (0)	
**Liver disease - moderate to severe (%)**		
Cases	9,745 (1.94)	
Controls	492,435 (98.06)	
Counts	502,180 (100)	
Missings	0 (0)	
**Myocardial infarction (%)**		
Cases	26,778 (5.33)	
Controls	475,402 (94.67)	
Counts	502,180 (100)	
Missings	0 (0)	
**Peptic ulcer (%)**		
Cases	56,085 (11.17)	
Controls	446,095 (88.83)	
Counts	502,180 (100)	
Missings	0 (0)	
**Peripheral vascular disease (%)**		
Cases	14,597 (2.91)	
Controls	487,583 (97.09)	
Counts	502,180 (100)	
Missings	0 (0)	
**Rheumatoid arthritis (%)**		
Cases	16,870 (3.36)	
Controls	485,310 (96.64)	
Counts	502,180 (100)	
Missings	0 (0)	

Supplementary Table 11. Pearson correlation between variants. Only those with r > 0.5 and r < -0.5 are shown.

Variable 1	Variable 2	R	Selected variable
Apolipoprotein B	LDL Direct	0.96	LDL Direct
Cholesterol	LDL Direct	0.95	LDL Direct
Apolipoprotein A	HDL cholesterol	0.92	HDL-cholesterol
Direct bilirubin	Total bilirubin	0.91	Total bilirubin
Apolipoprotein B	Cholesterol	0.89	Cholesterol
Asthma	COPD	0.84	COPD
Alanine aminotransferase	Aspartate aminotransferase	0.75	Alanine aminotransferase
Liver disease - mild	Liver disease – moderate to severe	0.66	Liver disease – moderate to severe
Alanine aminostransferase	Gamma glutamyltransferase	0.54	Alanine aminostransferase
Creatinine	Cystatin C	0.52	Cystatin C
Creatinine	Urate	0.52	Urate
Glucose	HbA1c	0.51	HbA1c
Creatinine	Testosterone	0.51	Creatinine
Calcium	Total protein	0.50	Calcium
Albumin	Calcium	0.50	Calcium

Supplementary Figure 1. Flow chart of the study participants. (A) Flowchart of the Long COVID cohorts. (B) Flowchart of the PACS cohort.

Note 1. "*Restrict to participants that answered the health and well-being web-questionnaire*" refers to including only UK Biobank participants who completed the Health and Well-being study.

Note 2. "*Restrict to participants that answered yes/no to all the questions from the health and well-being web-questionnaire*" means excluding those who answered "Do not know" or "Prefer not to answer" for any symptoms-related question.

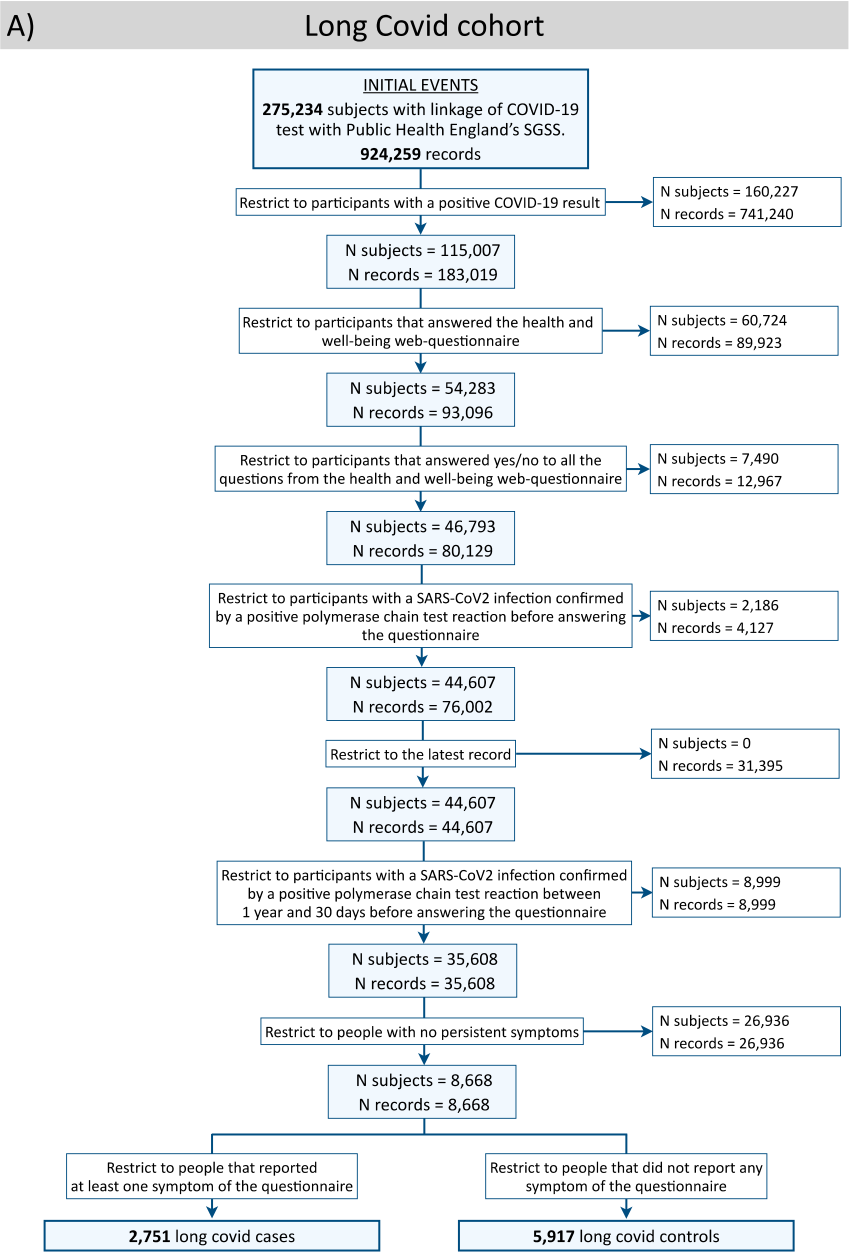

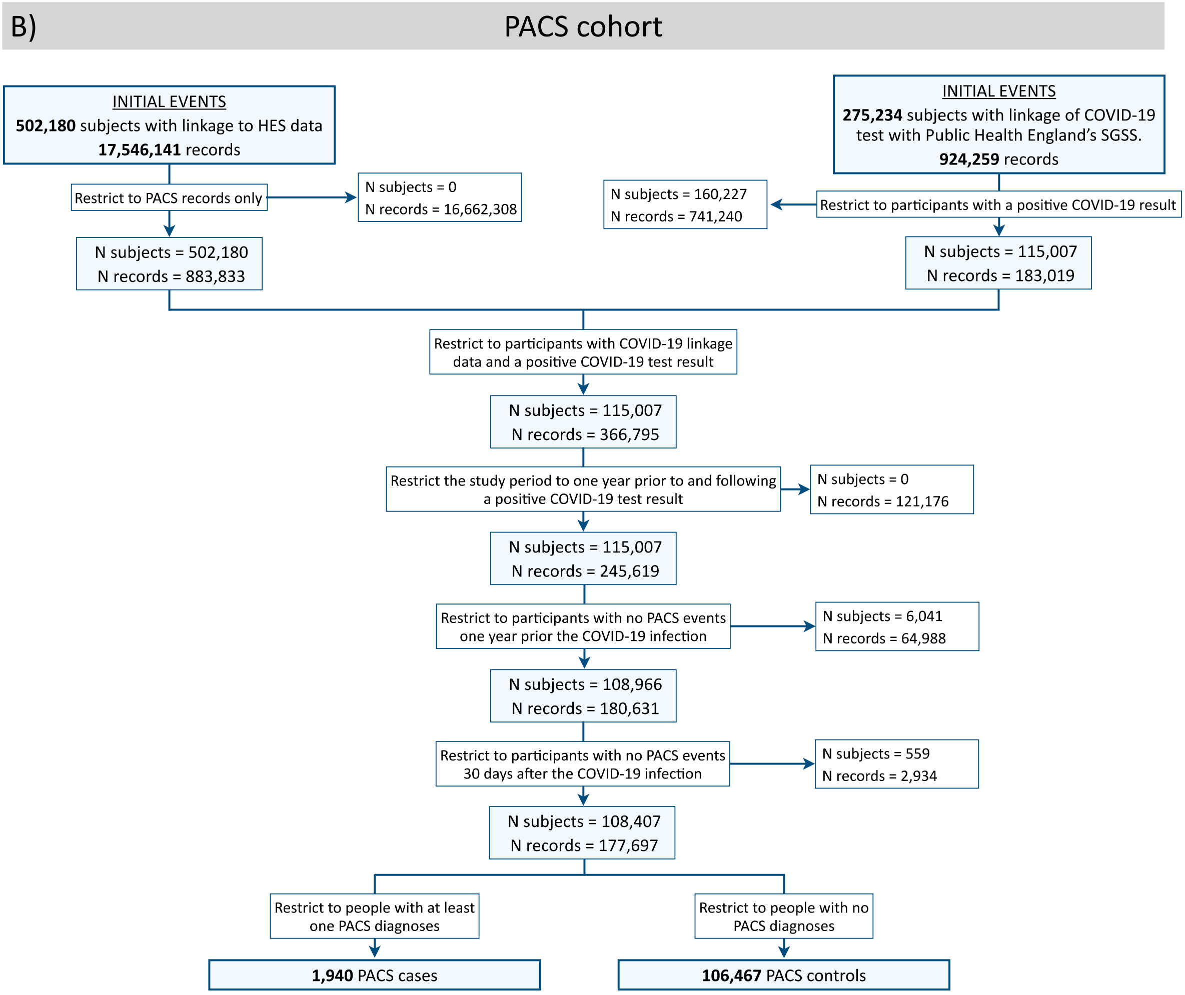
B)
PACS cohort
INITIAL EVENTS
502,180 subjects with linkage to HES data
17,546,141 records
Restrict to PACS records only
N subjects = 0
N records = 16,662,308
N subjects = 502,180
N records = 883,833
INITIAL EVENTS
275,234 subjects with linkage of COVID-19 test with Public Health England's SGSS.
924,259 records
Restrict to participants with a positive COVID-19 result
N subjects = 160,227
N records = 741,240
N subjects = 115,007
N records = 183,019
Restrict to participants with COVID-19 linkage data and a positive COVID-19 test result
N subjects = 115,007
N records = 366,795
Restrict the study period to one year prior to and following a positive COVID-19 test result
N subjects = 0
N records = 121,176
N subjects = 115,007
N records = 245,619
Restrict to participants with no PACS events one year prior the COVID-19 infection
N subjects = 6,041
N records = 64,988
N subjects = 108,966
N records = 180,631
Restrict to participants with no PACS events 30 days after the COVID-19 infection
N subjects = 559
N records = 2,934
N subjects = 108,407
N records = 177,697
Restrict to people with at least one PACS diagnoses
Restrict to people with no PACS diagnoses
1,940 PACS cases
106,467 PACS controls

Supplementary Figure 2. Pearson's correlation heatmap. (A) Correlation among sociodemographic factors. (B) Correlation among biomarkers. (C) Correlation among comorbidities.

A)

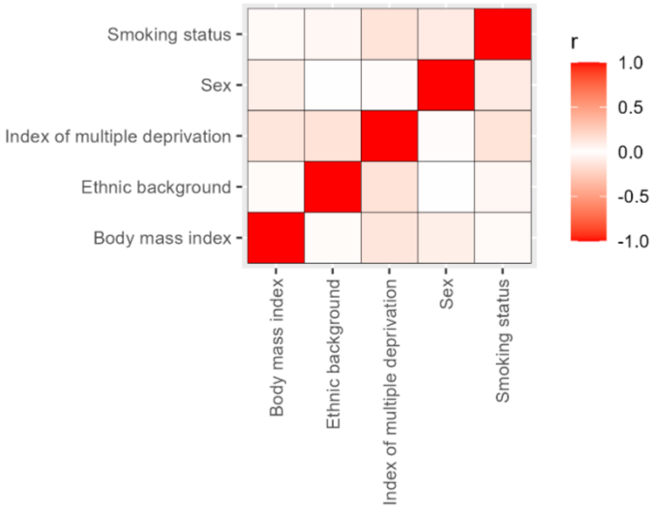

B)

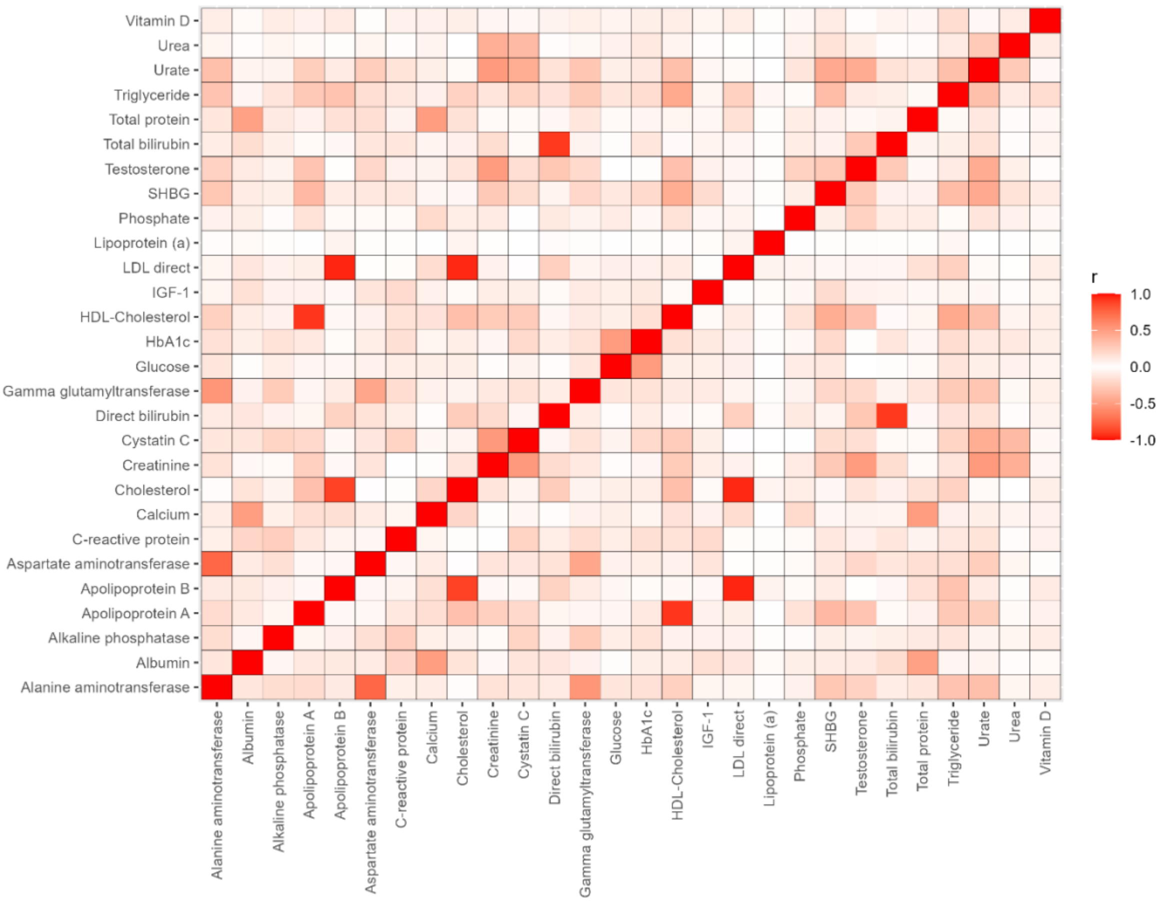

C)

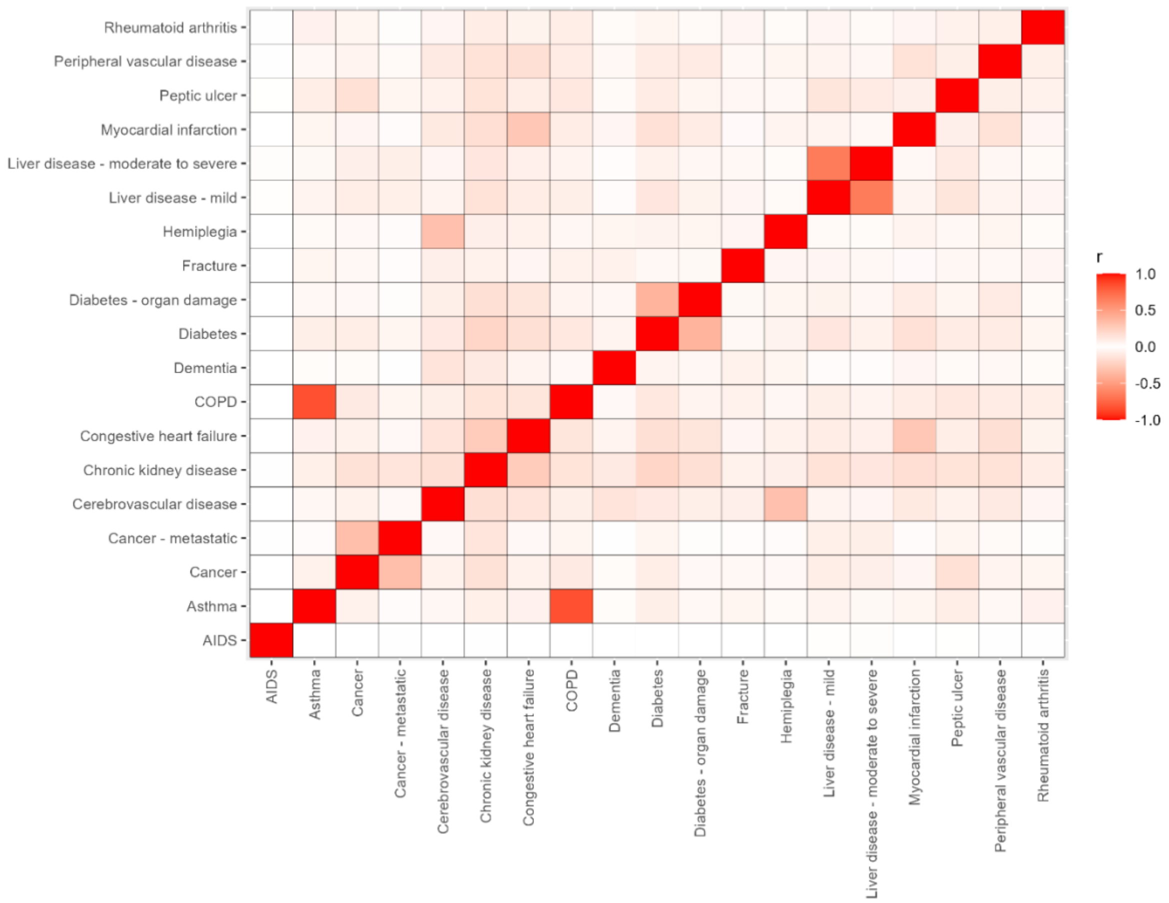
Rheumatoid arthritis
Peripheral vascular disease
Peptic ulcer
Myocardial infarction
Liver disease - moderate to severe
Liver disease - mild
Hemiplegia
Fracture
Diabetes - organ damage
Diabetes
Dementia
COPD
Congestive heart failure
Chronic kidney disease
Cerebrovascular disease
Cancer - metastatic
Cancer
Asthma
AIDS
r
1.0
0.5
0.0
-0.5
-1.0

Supplementary Figure 3. Long COVID sensitivity analysis. Forest plot of the adjusted analysis removing alanine aminotransferase, alkaline phosphatase, aspartate aminotransferase, gamma glutamyltransferase, HbA1c, glucose and IGF-1. Long COVID cohort included 8,668 participants, with 2,751 (32%) classified as cases and 5,917 (68%) as controls. Data is presented as odds ratio (OR) with 95% confidence intervals (CI). OR was calculated using a logistic regression. The two-sided p-value was calculated using ANOVA. (A) Results including only baseline characteristics risk factors. Dark blue corresponds to crude findings; light blue corresponds to adjusted findings. (B) Results including only biomarkers results. Dark maroon corresponds to crude findings; light maroon corresponds to adjusted findings. (C) Results including only comorbidities results. Orange corresponds to crude findings; yellow corresponds to adjusted findings.

A) Sociodemographic factors		OR (95% CI)	P-Value
Age when infected (Ref: <55)	55 to 59	0.94 (0.73, 1.21)	6.2e-01
	60 to 64	0.82 (0.64, 1.05)	1.1e-01
	65 to 69	0.76 (0.59, 0.97)	< 0.05
	70 to 74	0.77 (0.6, 1)	< 0.05
	>=75	0.83 (0.64, 1.08)	1.7e-01
Body mass index (Ref: Average)	Overweight	1.09 (0.98, 1.22)	1.2e-01
	Obesity	1.22 (1.04, 1.44)	< 0.05
Ethnic background (Ref: White)	Non-white	1.14 (0.9, 1.43)	2.8e-01
Index of multiple deprivation (Ref: Very affluent)	Affluent	1.07 (0.92, 1.24)	3.6e-01
	Average	1.16 (1.01, 1.35)	< 0.05
	Deprived	1.18 (1.02, 1.37)	< 0.05
	Very deprived	1.41 (1.22, 1.63)	< 0.05
Sex (Ref: Women)	Male	0.75 (0.66, 0.86)	< 0.05
Smoking status (Ref: Never)	Former	1.14 (1.03, 1.26)	< 0.05
	Current	1.05 (0.86, 1.28)	6.4e-01

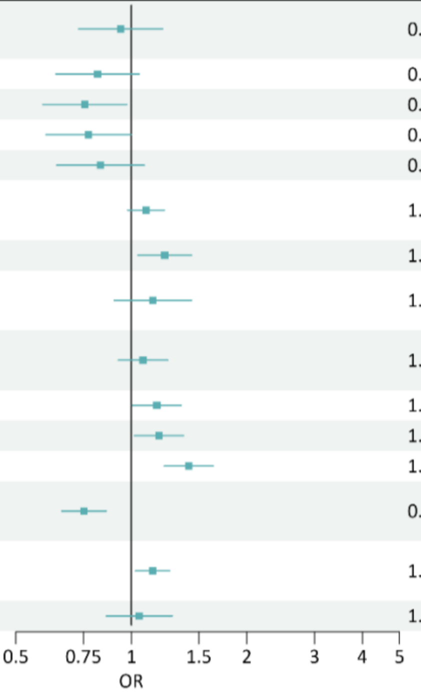

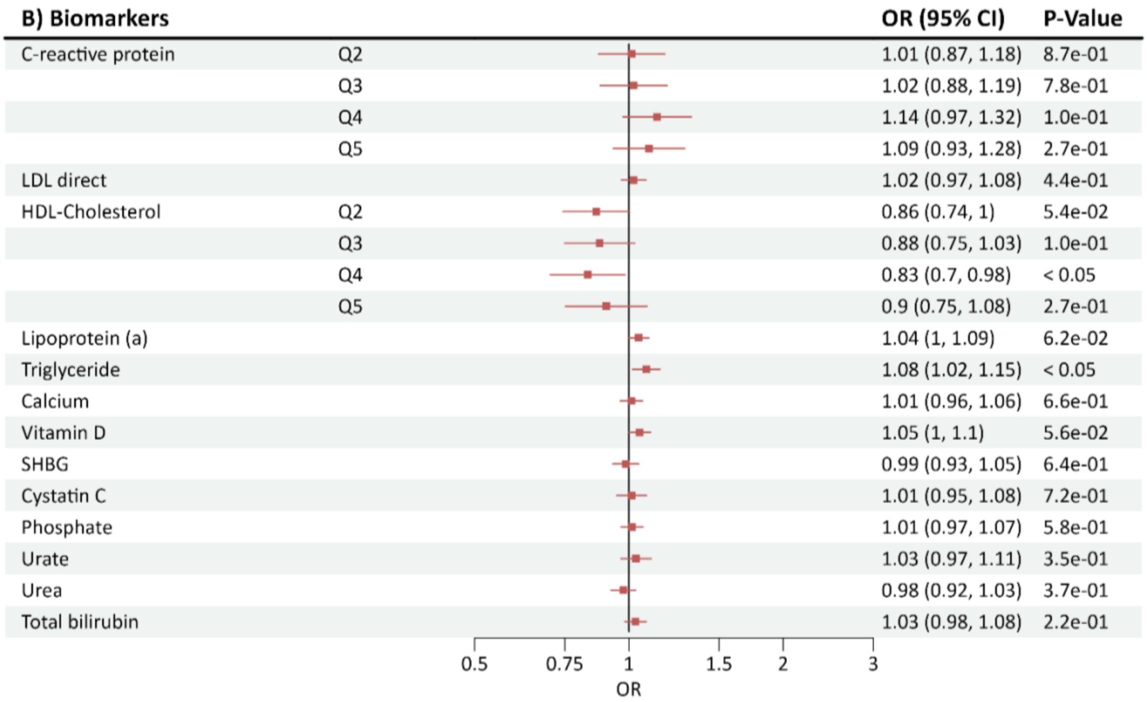
B) Biomarkers
OR (95% CI)
P-Value
C-reactive protein Q2 1.01 (0.87, 1.18) 8.7e-01
Q3 1.02 (0.88, 1.19) 7.8e-01
Q4 1.14 (0.97, 1.32) 1.0e-01
Q5 1.09 (0.93, 1.28) 2.7e-01
LDL direct 1.02 (0.97, 1.08) 4.4e-01
HDL-Cholesterol Q2 0.86 (0.74, 1) 5.4e-02
Q3 0.88 (0.75, 1.03) 1.0e-01
Q4 0.83 (0.7, 0.98) < 0.05
Q5 0.9 (0.75, 1.08) 2.7e-01
Lipoprotein (a) 1.04 (1, 1.09) 6.2e-02
Triglyceride 1.08 (1.02, 1.15) < 0.05
Calcium 1.01 (0.96, 1.06) 6.6e-01
Vitamin D 1.05 (1, 1.1) 5.6e-02
SHBG 0.99 (0.93, 1.05) 6.4e-01
Cystatin C 1.01 (0.95, 1.08) 7.2e-01
Phosphate 1.01 (0.97, 1.07) 5.8e-01
Urate 1.03 (0.97, 1.11) 3.5e-01
Urea 0.98 (0.92, 1.03) 3.7e-01
Total bilirubin 1.03 (0.98, 1.08) 2.2e-01
0.5
0.75
1
1.5
2
3
OR

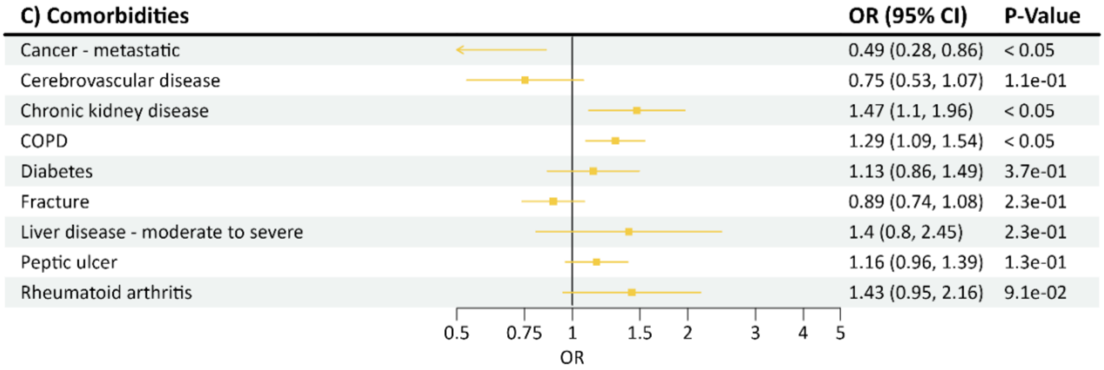
C) Comorbidities
OR (95% CI)
P-Value
Cancer - metastatic 0.49 (0.28, 0.86) < 0.05
Cerebrovascular disease 0.75 (0.53, 1.07) 1.1e-01
Chronic kidney disease 1.47 (1.1, 1.96) < 0.05
COPD 1.29 (1.09, 1.54) < 0.05
Diabetes 1.13 (0.86, 1.49) 3.7e-01
Fracture 0.89 (0.74, 1.08) 2.3e-01
Liver disease - moderate to severe 1.4 (0.8, 2.45) 2.3e-01
Peptic ulcer 1.16 (0.96, 1.39) 1.3e-01
Rheumatoid arthritis 1.43 (0.95, 2.16) 9.1e-02
0.5
0.75
1
1.5
2
3
4
5
OR

Supplementary Figure 4. PACS sensitivity analysis. Forest plot of the adjusted analysis removing alanine aminotransferase, alkaline phosphatase, aspartate aminotransferase, gamma glutamyltransferase, hbA1c, glucose and IGF-1. PACS cohort included 108,407 participants, with 1,940 (2%) classified as cases and 106,467 classified as controls (98%). Data is presented as odds ratio (OR) with 95% confidence intervals (CI). OR was calculated using a logistic regression. The two-sided p-value was calculated using ANOVA. (A) Results including only baseline characteristics risk factors. Dark blue corresponds to crude findings; light blue corresponds to adjusted findings. (B) Results including only biomarkers results. Dark maroon corresponds to crude findings; light maroon corresponds to adjusted findings. (C) Results including only comorbidities results. Orange corresponds to crude findings; yellow corresponds to adjusted findings.

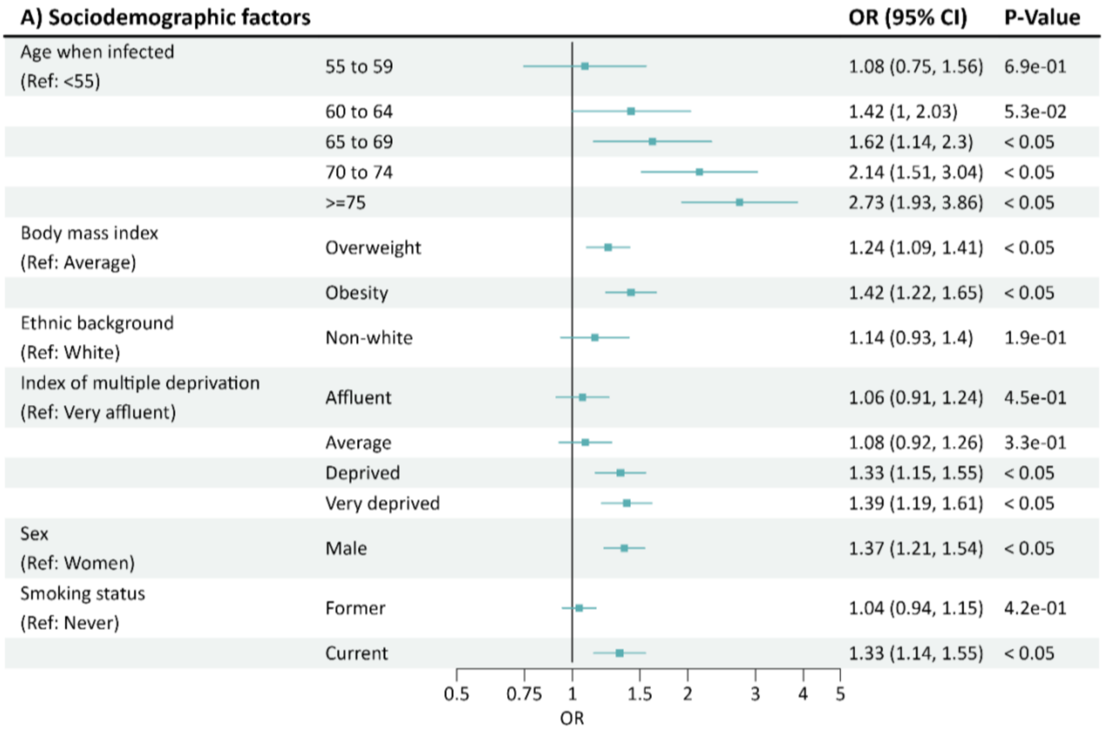

B) Biomarkers

		OR (95% CI)	P-Value
C-reactive protein	Q2	0.96 (0.81, 1.15)	6.7e-01
	Q3	1.03 (0.87, 1.22)	7.0e-01
	Q4	1.18 (1, 1.39)	5.2e-02
	Q5	1.25 (1.06, 1.48)	< 0.05
LDL direct	Q2	0.94 (0.82, 1.08)	3.8e-01
	Q3	0.82 (0.71, 0.95)	< 0.05
	Q4	0.88 (0.76, 1.02)	9.2e-02
	Q5	0.88 (0.75, 1.02)	9.2e-02
HDL-Cholesterol	Q2	1.04 (0.91, 1.19)	5.8e-01
	Q3	1.05 (0.91, 1.22)	5.2e-01
	Q4	1.04 (0.88, 1.22)	6.6e-01
	Q5	1.01 (0.84, 1.22)	9.0e-01
Lipoprotein (a)		1.05 (1, 1.1)	< 0.05
Triglyceride	Q2	0.96 (0.81, 1.13)	5.9e-01
	Q3	0.99 (0.83, 1.17)	8.7e-01
	Q4	0.99 (0.83, 1.17)	8.7e-01
	Q5	0.94 (0.78, 1.13)	5.0e-01
Calcium	Q2	0.89 (0.77, 1.03)	1.2e-01
	Q3	0.89 (0.77, 1.03)	1.1e-01
	Q4	0.91 (0.78, 1.05)	2.1e-01
	Q5	0.98 (0.85, 1.14)	7.9e-01
Vitamin D		1.04 (0.99, 1.09)	1.5e-01
SHBG		1 (0.94, 1.07)	9.7e-01
Cystatin C		1.09 (1.04, 1.15)	< 0.05
Phosphate		1.05 (1, 1.1)	6.1e-02
Urate	Q2	1.03 (0.86, 1.24)	7.2e-01
	Q3	1.14 (0.96, 1.37)	1.4e-01
	Q4	1.08 (0.9, 1.3)	4.2e-01
	Q5	1.08 (0.89, 1.32)	4.3e-01
Urea	Q2	1.04 (0.88, 1.22)	6.7e-01
	Q3	1.01 (0.86, 1.18)	9.4e-01
	Q4	0.98 (0.84, 1.15)	8.2e-01
	Q5	1.01 (0.86, 1.19)	9.1e-01

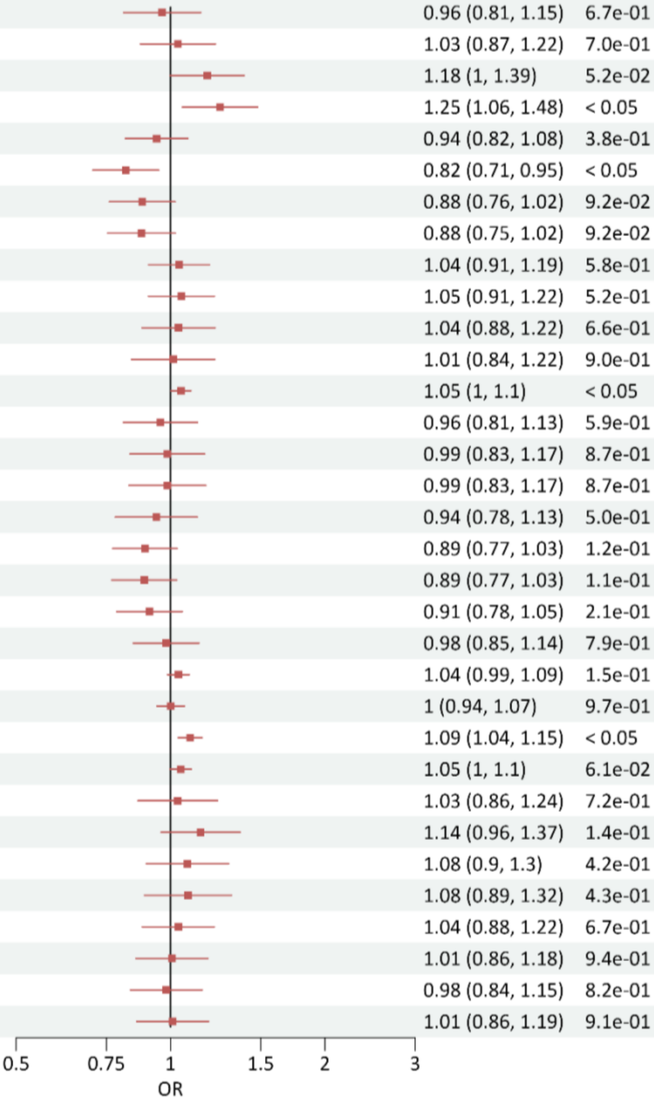

C) Comorbidities

	OR (95% CI)	P-Value
Cancer	1.18 (1.06, 1.3)	< 0.05
Cancer - metastatic	1.54 (1.21, 1.95)	< 0.05
Cerebrovascular disease	1.09 (0.91, 1.3)	3.5e-01
Congestive heart failure	2.56 (2.12, 3.08)	< 0.05
Chronic kidney disease	1.35 (1.17, 1.56)	< 0.05
COPD	1.43 (1.27, 1.6)	< 0.05
Dementia	1.33 (1, 1.78)	5.3e-02
Diabetes	1.54 (1.34, 1.76)	< 0.05
Fracture	1.1 (0.94, 1.28)	2.3e-01
Hemiplegia	1.34 (0.95, 1.88)	9.8e-02
Liver disease - moderate to severe	1.27 (0.96, 1.68)	1.0e-01
Myocardial infarction	2.35 (2.03, 2.73)	< 0.05
Peptic ulcer	1.43 (1.26, 1.61)	< 0.05
Peripheral vascular disease	1.47 (1.2, 1.81)	< 0.05
Rheumatoid arthritis	1.54 (1.27, 1.85)	< 0.05

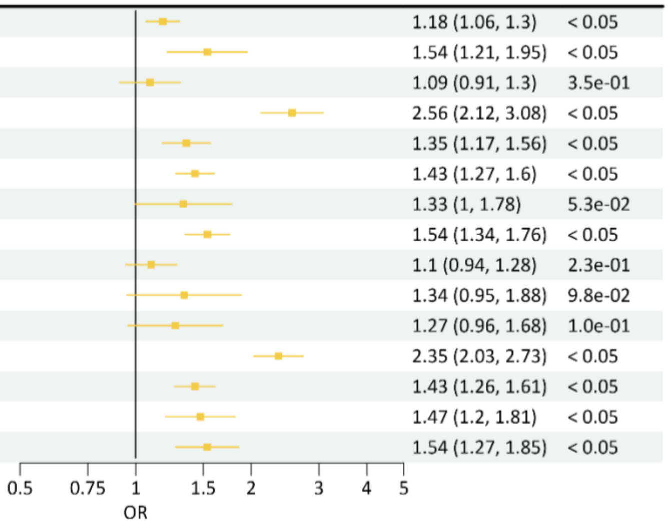

Supplementary Figure 5. Sex-stratified analysis for Long COVID. Long COVID cohort included 8,668 participants, with 2,751 (32%) classified as cases and 5,917 (68%) as controls. Data is presented as odds ratio (OR) with 95% confidence intervals (CI). OR was calculated using a logistic regression. The two-sided p-value was calculated using ANOVA. (A) Results including only baseline characteristics risk factors. Dark blue corresponds to crude findings; light blue corresponds to adjusted findings. (B) Results including only biomarkers results. Dark maroon corresponds to crude findings; light maroon corresponds to adjusted findings. (C) Results including only comorbidities results. Orange corresponds to crude findings; yellow corresponds to adjusted findings.

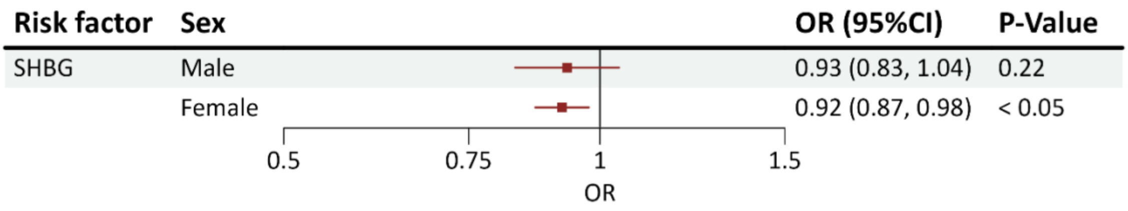

Supplementary Figure 6. Sex-stratified analysis for PACS. PACS cohort included 108,407 participants, with 1,940 (2%) classified as cases and 106,467 classified as controls (98%). Data is presented as odds ratio (OR) with 95% confidence intervals (CI). OR was calculated using a logistic regression. The two-sided p-value was calculated using ANOVA. (A) Results including only baseline characteristics risk factors. Dark blue corresponds to crude findings; light blue corresponds to adjusted findings. (B) Results including only biomarkers results. Dark maroon corresponds to crude findings; light maroon corresponds to adjusted findings. (C) Results including only comorbidities results. Orange corresponds to crude findings; yellow corresponds to adjusted findings.

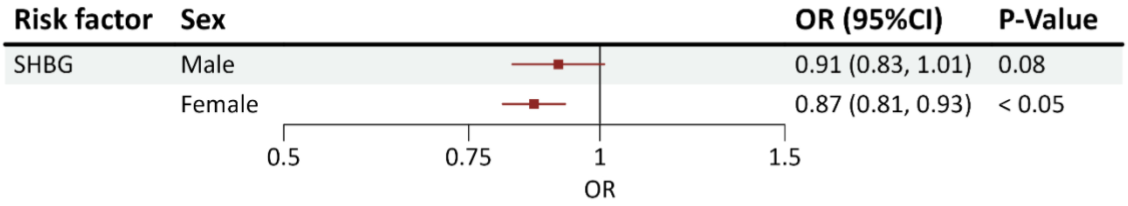

Supplementary Figure 7. Long COVID definition (requiring at least three symptoms) sensitivity analysis. Long COVID cohort included 8,673 participants, with 594 (3%) classified as cases and 8,079 (97%) as controls. Data is presented as odds ratio (OR) with 95% confidence intervals (CI). OR was calculated using a logistic regression. The two-sided p-value was calculated using ANOVA. (A) Results including only baseline characteristics risk factors. Dark blue corresponds to crude findings; light blue corresponds to adjusted findings. (B) Results including only biomarkers results. Dark maroon corresponds to crude findings; light maroon corresponds to adjusted findings. (C) Results including only comorbidities results. Orange corresponds to crude findings; yellow corresponds to adjusted findings.

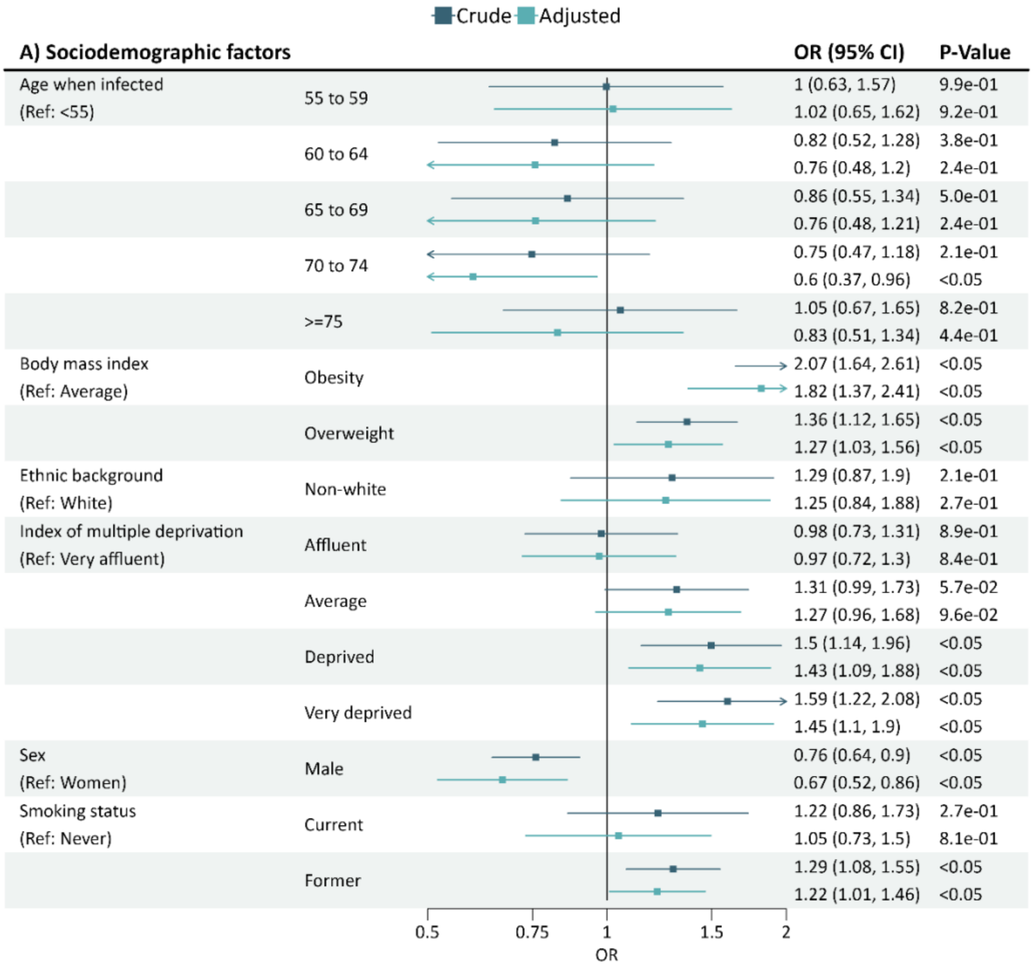

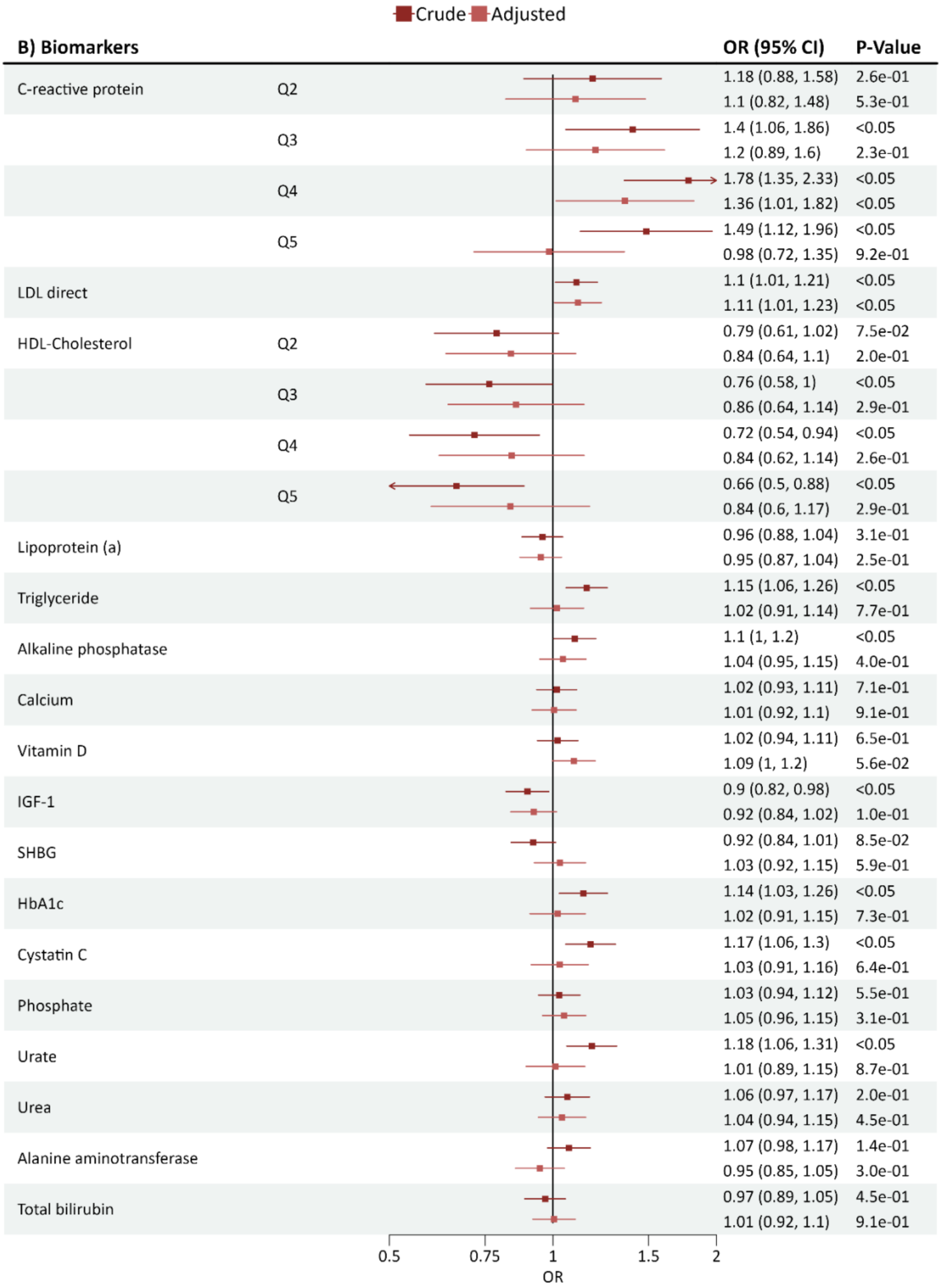
Crude
Adjusted
B) Biomarkers
OR (95% CI)
P-Value
C-reactive protein
Q2
1.18 (0.88, 1.58) 2.6e-01
1.1 (0.82, 1.48) 5.3e-01
Q3
1.4 (1.06, 1.86) <0.05
1.2 (0.89, 1.6) 2.3e-01
Q4
1.78 (1.35, 2.33) <0.05
1.36 (1.01, 1.82) <0.05
Q5
1.49 (1.12, 1.96) <0.05
0.98 (0.72, 1.35) 9.2e-01
LDL direct
1.1 (1.01, 1.21) <0.05
1.11 (1.01, 1.23) <0.05
HDL-Cholesterol
Q2
0.79 (0.61, 1.02) 7.5e-02
0.84 (0.64, 1.1) 2.0e-01
Q3
0.76 (0.58, 1) <0.05
0.86 (0.64, 1.14) 2.9e-01
Q4
0.72 (0.54, 0.94) <0.05
0.84 (0.62, 1.14) 2.6e-01
Q5
0.66 (0.5, 0.88) <0.05
0.84 (0.6, 1.17) 2.9e-01
Lipoprotein (a)
0.96 (0.88, 1.04) 3.1e-01
0.95 (0.87, 1.04) 2.5e-01
Triglyceride
1.15 (1.06, 1.26) <0.05
1.02 (0.91, 1.14) 7.7e-01
Alkaline phosphatase
1.1 (1, 1.2) <0.05
1.04 (0.95, 1.15) 4.0e-01
Calcium
1.02 (0.93, 1.11) 7.1e-01
1.01 (0.92, 1.1) 9.1e-01
Vitamin D
1.02 (0.94, 1.11) 6.5e-01
1.09 (1, 1.2) 5.6e-02
IGF-1
0.9 (0.82, 0.98) <0.05
0.92 (0.84, 1.02) 1.0e-01
SHBG
0.92 (0.84, 1.01) 8.5e-02
1.03 (0.92, 1.15) 5.9e-01
HbA1c
1.14 (1.03, 1.26) <0.05
1.02 (0.91, 1.15) 7.3e-01
Cystatin C
1.17 (1.06, 1.3) <0.05
1.03 (0.91, 1.16) 6.4e-01
Phosphate
1.03 (0.94, 1.12) 5.5e-01
1.05 (0.96, 1.15) 3.1e-01
Urate
1.18 (1.06, 1.31) <0.05
1.01 (0.89, 1.15) 8.7e-01
Urea
1.06 (0.97, 1.17) 2.0e-01
1.04 (0.94, 1.15) 4.5e-01
Alanine aminotransferase
1.07 (0.98, 1.17) 1.4e-01
0.95 (0.85, 1.05) 3.0e-01
Total bilirubin
0.97 (0.89, 1.05) 4.5e-01
1.01 (0.92, 1.1) 9.1e-01
0.5
0.75
1
1.5
2
OR

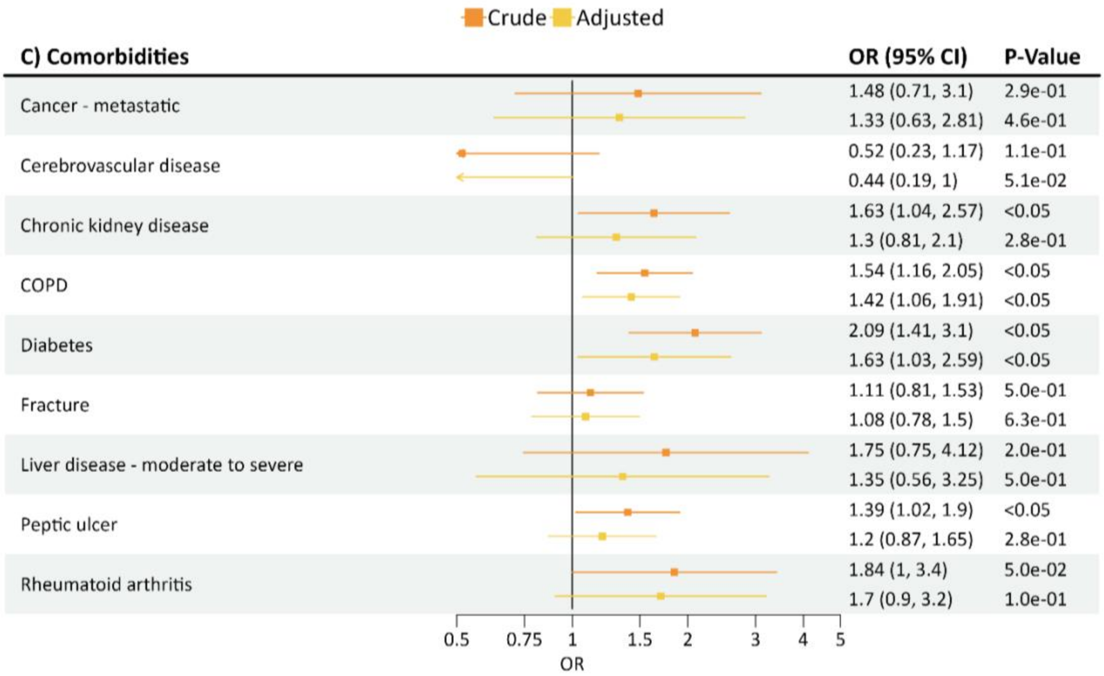

Crude
Adjusted
C) Comorbidities
OR (95% CI)
P-Value
Cancer - metastatic
1.48 (0.71, 3.1) 2.9e-01
1.33 (0.63, 2.81) 4.6e-01
Cerebrovascular disease
0.52 (0.23, 1.17) 1.1e-01
0.44 (0.19, 1) 5.1e-02
Chronic kidney disease
1.63 (1.04, 2.57) <0.05
1.3 (0.81, 2.1) 2.8e-01
COPD
1.54 (1.16, 2.05) <0.05
1.42 (1.06, 1.91) <0.05
Diabetes
2.09 (1.41, 3.1) <0.05
1.63 (1.03, 2.59) <0.05
Fracture
1.11 (0.81, 1.53) 5.0e-01
1.08 (0.78, 1.5) 6.3e-01
Liver disease - moderate to severe
1.75 (0.75, 4.12) 2.0e-01
1.35 (0.56, 3.25) 5.0e-01
Peptic ulcer
1.39 (1.02, 1.9) <0.05
1.2 (0.87, 1.65) 2.8e-01
Rheumatoid arthritis
1.84 (1, 3.4) 5.0e-02
1.7 (0.9, 3.2) 1.0e-01
0.5 0.75 1 1.5 2 3 4 5
OR

Supplementary Figure 8. Long COVID definition (requiring at least three months after infection) sensitivity analysis. Long COVID cohort included 7,669 participants, with 2,383 (31%) classified as cases and 5,286 (69%) as controls. Data is presented as odds ratio (OR) with 95% confidence intervals (CI). OR was calculated using a logistic regression. The two-sided p-value was calculated using ANOVA. (A) Results including only baseline characteristics risk factors. Dark blue corresponds to crude findings; light blue corresponds to adjusted findings. (B) Results including only biomarkers results. Dark maroon corresponds to crude findings; light maroon corresponds to adjusted findings. (C) Results including only comorbidities results. Orange corresponds to crude findings; yellow corresponds to adjusted findings.

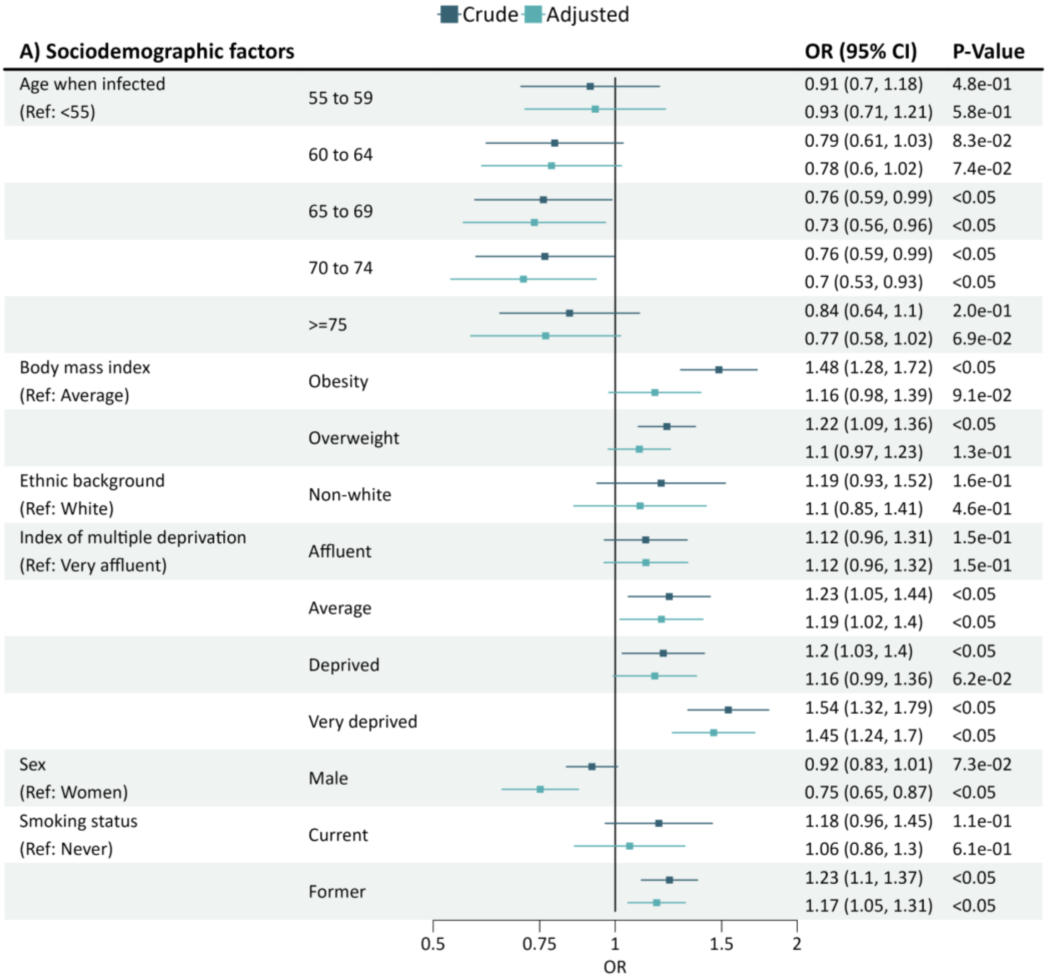

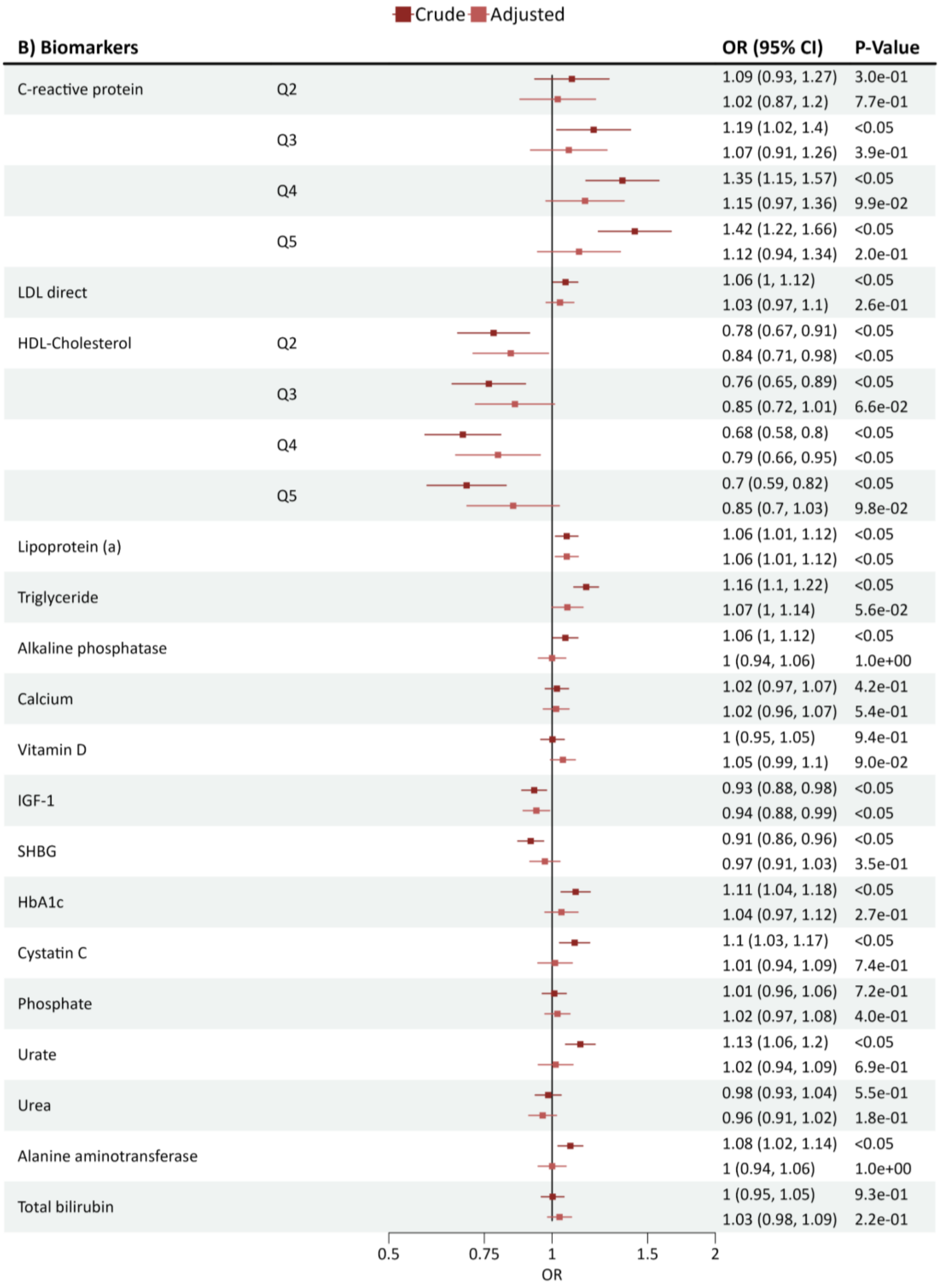

B) Biomarkers		OR (95% CI)	P-Value
C-reactive protein	Q2	1.09 (0.93, 1.27)	3.0e-01
		1.02 (0.87, 1.2)	7.7e-01
	Q3	1.19 (1.02, 1.4)	<0.05
		1.07 (0.91, 1.26)	3.9e-01
	Q4	1.35 (1.15, 1.57)	<0.05
		1.15 (0.97, 1.36)	9.9e-02
	Q5	1.42 (1.22, 1.66)	<0.05
		1.12 (0.94, 1.34)	2.0e-01
LDL direct		1.06 (1, 1.12)	<0.05
		1.03 (0.97, 1.1)	2.6e-01
HDL-Cholesterol	Q2	0.78 (0.67, 0.91)	<0.05
		0.84 (0.71, 0.98)	<0.05
	Q3	0.76 (0.65, 0.89)	<0.05
		0.85 (0.72, 1.01)	6.6e-02
	Q4	0.68 (0.58, 0.8)	<0.05
		0.79 (0.66, 0.95)	<0.05
	Q5	0.7 (0.59, 0.82)	<0.05
		0.85 (0.7, 1.03)	9.8e-02
Lipoprotein (a)		1.06 (1.01, 1.12)	<0.05
		1.06 (1.01, 1.12)	<0.05
Triglyceride		1.16 (1.1, 1.22)	<0.05
		1.07 (1, 1.14)	5.6e-02
Alkaline phosphatase		1.06 (1, 1.12)	<0.05
		1 (0.94, 1.06)	1.0e+00
Calcium		1.02 (0.97, 1.07)	4.2e-01
		1.02 (0.96, 1.07)	5.4e-01
Vitamin D		1 (0.95, 1.05)	9.4e-01
		1.05 (0.99, 1.1)	9.0e-02
IGF-1		0.93 (0.88, 0.98)	<0.05
		0.94 (0.88, 0.99)	<0.05
SHBG		0.91 (0.86, 0.96)	<0.05
		0.97 (0.91, 1.03)	3.5e-01
HbA1c		1.11 (1.04, 1.18)	<0.05
		1.04 (0.97, 1.12)	2.7e-01
Cystatin C		1.1 (1.03, 1.17)	<0.05
		1.01 (0.94, 1.09)	7.4e-01
Phosphate		1.01 (0.96, 1.06)	7.2e-01
		1.02 (0.97, 1.08)	4.0e-01
Urate		1.13 (1.06, 1.2)	<0.05
		1.02 (0.94, 1.09)	6.9e-01
Urea		0.98 (0.93, 1.04)	5.5e-01
		0.96 (0.91, 1.02)	1.8e-01
Alanine aminotransferase		1.08 (1.02, 1.14)	<0.05
		1 (0.94, 1.06)	1.0e+00
Total bilirubin		1 (0.95, 1.05)	9.3e-01
		1.03 (0.98, 1.09)	2.2e-01

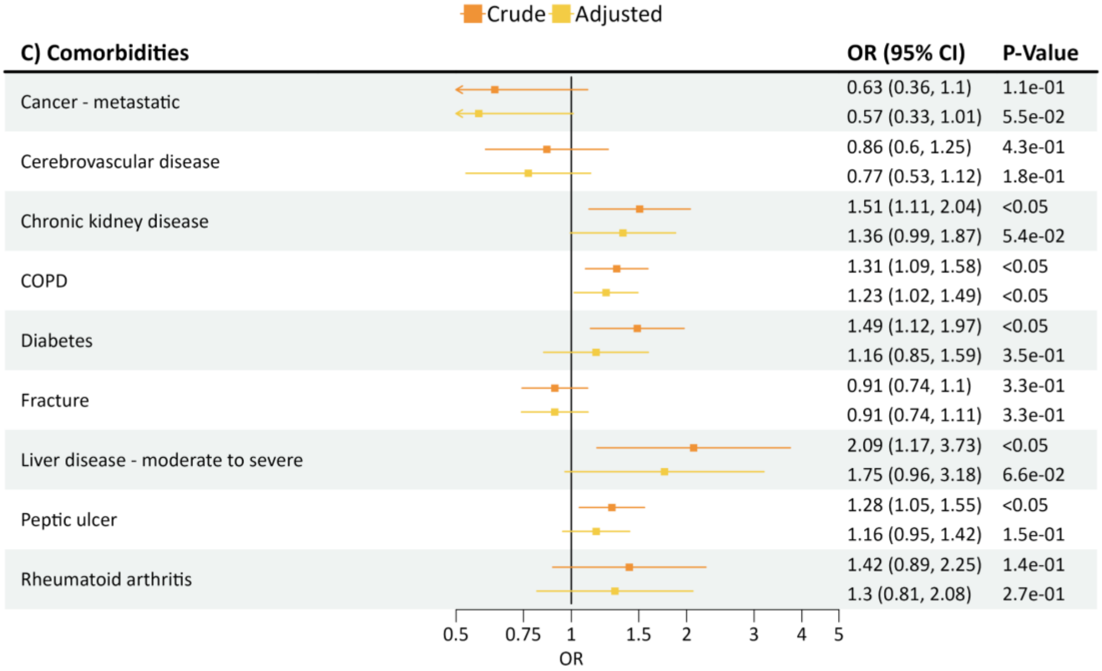
Crude
Adjusted
C) Comorbidities
OR (95% CI)
P-Value
Cancer - metastatic
0.63 (0.36, 1.1) 1.1e-01
0.57 (0.33, 1.01) 5.5e-02
Cerebrovascular disease
0.86 (0.6, 1.25) 4.3e-01
0.77 (0.53, 1.12) 1.8e-01
Chronic kidney disease
1.51 (1.11, 2.04) <0.05
1.36 (0.99, 1.87) 5.4e-02
COPD
1.31 (1.09, 1.58) <0.05
1.23 (1.02, 1.49) <0.05
Diabetes
1.49 (1.12, 1.97) <0.05
1.16 (0.85, 1.59) 3.5e-01
Fracture
0.91 (0.74, 1.1) 3.3e-01
0.91 (0.74, 1.11) 3.3e-01
Liver disease - moderate to severe
2.09 (1.17, 3.73) <0.05
1.75 (0.96, 3.18) 6.6e-02
Peptic ulcer
1.28 (1.05, 1.55) <0.05
1.16 (0.95, 1.42) 1.5e-01
Rheumatoid arthritis
1.42 (0.89, 2.25) 1.4e-01
1.3 (0.81, 2.08) 2.7e-01
0.5
0.75
1
1.5
2
3
4
5
OR

Supplementary Figure 9. Long COVID Bonferroni correction. Long COVID cohort included 8,668 participants, with 2,751 (32%) classified as cases and 5,917 (68%) as controls. Data is presented as odds ratio (OR) with 95% confidence intervals (CI). OR was calculated using a logistic regression. The two-sided p-value was calculated using ANOVA. (A) Results including only baseline characteristics risk factors. Dark blue corresponds to crude findings; light blue corresponds to adjusted findings. (B) Results including only biomarkers results. Dark maroon corresponds to crude findings; light maroon corresponds to adjusted findings. (C) Results including only comorbidities results. Orange corresponds to crude findings; yellow corresponds to adjusted findings.

A) Sociodemographic factors

		OR (Bonferroni CI)	P-Value
Age when infected (Ref: <55)	55 to 59	0.92 (0.6, 1.4)	5.0e-01
	60 to 64	0.79 (0.52, 1.2)	6.1e-02
	65 to 69	0.71 (0.47, 1.09)	9.8e-03
	70 to 74	0.72 (0.47, 1.11)	1.4e-02
	>=75	0.77 (0.49, 1.2)	5.1e-02
Body mass index (Ref: Average)	Overweight	1.09 (0.91, 1.31)	1.2e-01
	Obesity	1.2 (0.91, 1.57)	3.1e-02
Ethnic background (Ref: White)	Non-white	1.11 (0.75, 1.65)	3.6e-01
Index of multiple deprivation (Ref: Very affluent)	Affluent	1.08 (0.84, 1.38)	3.3e-01
	Average	1.16 (0.91, 1.49)	4.6e-02
	Deprived	1.18 (0.92, 1.51)	2.7e-02
	Very deprived	1.41 (1.1, 1.8)	< 0.0011
Sex (Ref: Women)	Male	0.75 (0.6, 0.94)	< 0.0011
Smoking status (Ref: Never)	Former	1.13 (0.96, 1.35)	1.6e-02
	Current	1.04 (0.74, 1.44)	7.3e-01

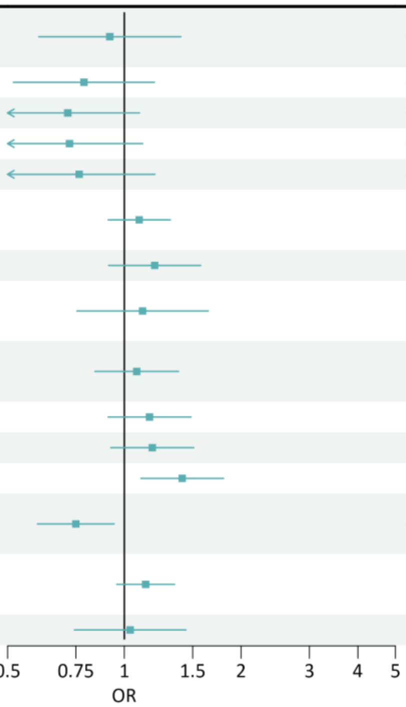

Bonferroni P-Value: 0.0011

B) Biomarkers

		OR (Bonferroni CI)	P-Value
C-reactive protein	Q2	1.01 (0.79, 1.3)	8.9e-01
	Q3	1.01 (0.78, 1.3)	9.0e-01
	Q4	1.11 (0.86, 1.44)	1.7e-01
	Q5	1.06 (0.81, 1.39)	4.9e-01
LDL direct		1.02 (0.93, 1.12)	4.8e-01
HDL-Cholesterol	Q2	0.86 (0.67, 1.11)	5.6e-02
	Q3	0.87 (0.67, 1.14)	9.7e-02
	Q4	0.83 (0.62, 1.09)	2.6e-02
	Q5	0.89 (0.66, 1.21)	2.3e-01
Lipoprotein (a)		1.05 (0.97, 1.13)	5.3e-02
Triglyceride		1.08 (0.97, 1.2)	1.7e-02
Alkaline phosphatase		0.99 (0.9, 1.08)	6.3e-01
Calcium		1.02 (0.94, 1.11)	4.9e-01
Vitamin D		1.05 (0.97, 1.14)	4.6e-02
IGF-1		0.93 (0.85, 1.02)	7.8e-03
SHBG		0.97 (0.88, 1.08)	3.6e-01
HbA1c		1.05 (0.94, 1.17)	1.5e-01
Cystatin C		1.02 (0.91, 1.14)	5.8e-01
Phosphate		1.01 (0.93, 1.1)	6.1e-01
Urate		1.03 (0.91, 1.15)	4.8e-01
Urea		0.98 (0.89, 1.07)	3.9e-01
Alanine aminotransferase		1 (0.91, 1.09)	9.1e-01
Total bilirubin		1.03 (0.96, 1.12)	1.6e-01

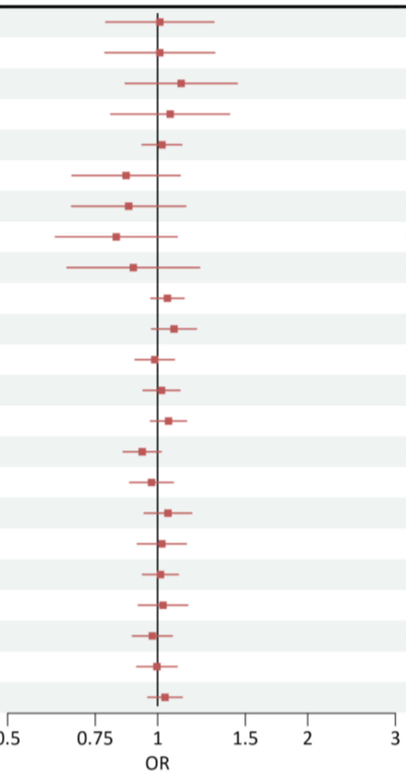

Bonferroni P-Value: 0.0011

C) Comorbidities	OR (Bonferroni CI)	P-Value
Cancer - metastatic	0.49 (0.19, 1.26)	1.4e-02
Cerebrovascular disease	0.76 (0.42, 1.36)	1.2e-01
Chronic kidney disease	1.48 (0.91, 2.39)	7.8e-03
COPD	1.29 (0.96, 1.73)	4.5e-03
Diabetes	1.04 (0.63, 1.7)	8.0e-01
Fracture	0.89 (0.65, 1.22)	2.4e-01
Liver disease - moderate to severe	1.38 (0.55, 3.5)	2.6e-01
Peptic ulcer	1.17 (0.85, 1.59)	1.1e-01
Rheumatoid arthritis	1.44 (0.72, 2.88)	8.3e-02

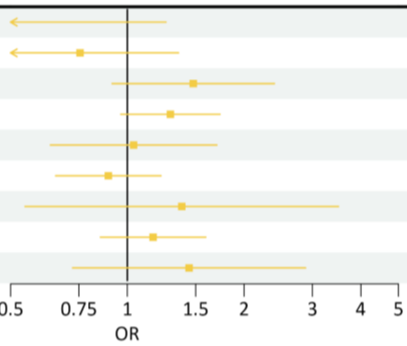

Bonferroni P-Value: 0.0011

Supplementary Figure 10. PACS Bonferroni correction. PACS cohort included 108,407 participants, with 1,940 (2%) classified as cases and 106,467 classified as controls (98%). Data is presented as odds ratio (OR) with 95% confidence intervals (CI). OR was calculated using a logistic regression. The two-sided p-value was calculated using ANOVA. (A) Results including only baseline characteristics risk factors. Dark blue corresponds to crude findings; light blue corresponds to adjusted findings. (B) Results including only biomarkers results. Dark maroon corresponds to crude findings; light maroon corresponds to adjusted findings. (C) Results including only comorbidities results. Orange corresponds to crude findings; yellow corresponds to adjusted findings.

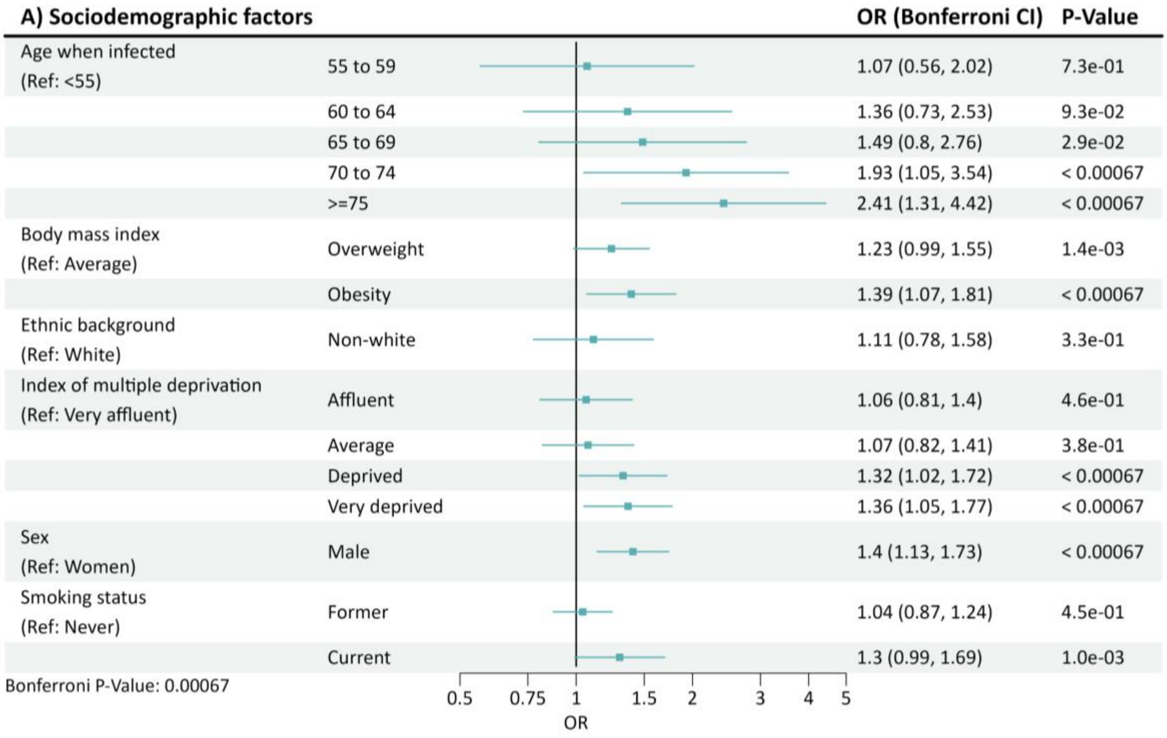

B) Biomarkers

		OR (Bonferroni CI)	P-Value
C-reactive protein	Q2	0.94 (0.7, 1.28)	5.2e-01
	Q3	1 (0.74, 1.34)	9.8e-01
	Q4	1.12 (0.83, 1.49)	2.0e-01
	Q5	1.15 (0.85, 1.55)	1.2e-01
LDL direct	Q2	0.94 (0.74, 1.2)	4.1e-01
	Q3	0.82 (0.63, 1.06)	9.4e-03
	Q4	0.88 (0.68, 1.14)	9.4e-02
	Q5	0.87 (0.67, 1.14)	8.2e-02
HDL-Cholesterol	Q2	1.04 (0.82, 1.32)	5.7e-01
	Q3	1.05 (0.82, 1.36)	4.8e-01
	Q4	1.04 (0.78, 1.38)	6.4e-01
	Q5	1.02 (0.73, 1.41)	8.7e-01
Lipoprotein (a)		1.05 (0.97, 1.13)	3.9e-02
Triglyceride	Q2	0.95 (0.71, 1.27)	5.6e-01
	Q3	0.98 (0.73, 1.31)	7.7e-01
	Q4	0.97 (0.72, 1.31)	7.2e-01
	Q5	0.92 (0.67, 1.26)	3.5e-01
Alkaline phosphatase	Q2	1.37 (1.02, 1.82)	< 0.00067
	Q3	1.27 (0.95, 1.69)	5.5e-03
	Q4	1.21 (0.9, 1.62)	2.6e-02
	Q5	1.35 (1.01, 1.79)	< 0.00067
Calcium	Q2	0.89 (0.7, 1.15)	1.3e-01
	Q3	0.89 (0.69, 1.15)	1.2e-01
	Q4	0.91 (0.7, 1.18)	2.1e-01
	Q5	0.98 (0.76, 1.27)	7.8e-01
Vitamin D		1.04 (0.96, 1.14)	8.2e-02
IGF-1	Q2	0.91 (0.72, 1.14)	1.4e-01
	Q3	0.84 (0.65, 1.07)	1.4e-02
	Q4	0.85 (0.66, 1.1)	3.5e-02
	Q5	0.84 (0.64, 1.1)	2.8e-02
SHBG		1 (0.89, 1.12)	9.6e-01
HbA1c	Q2	1.06 (0.78, 1.45)	5.2e-01
	Q3	1.26 (0.93, 1.69)	8.6e-03
	Q4	1.2 (0.89, 1.61)	3.6e-02
	Q5	1.29 (0.96, 1.74)	3.0e-03
Cystatin C		1.09 (0.99, 1.2)	1.8e-03
Phosphate		1.05 (0.96, 1.14)	6.5e-02
Urate	Q2	1.02 (0.75, 1.39)	8.3e-01
	Q3	1.13 (0.83, 1.54)	1.8e-01
	Q4	1.06 (0.77, 1.47)	5.2e-01
	Q5	1.07 (0.76, 1.5)	5.3e-01
Urea	Q2	1.04 (0.78, 1.38)	6.4e-01
	Q3	1.01 (0.76, 1.34)	9.2e-01
	Q4	0.98 (0.74, 1.3)	8.3e-01
	Q5	1.02 (0.77, 1.34)	8.4e-01

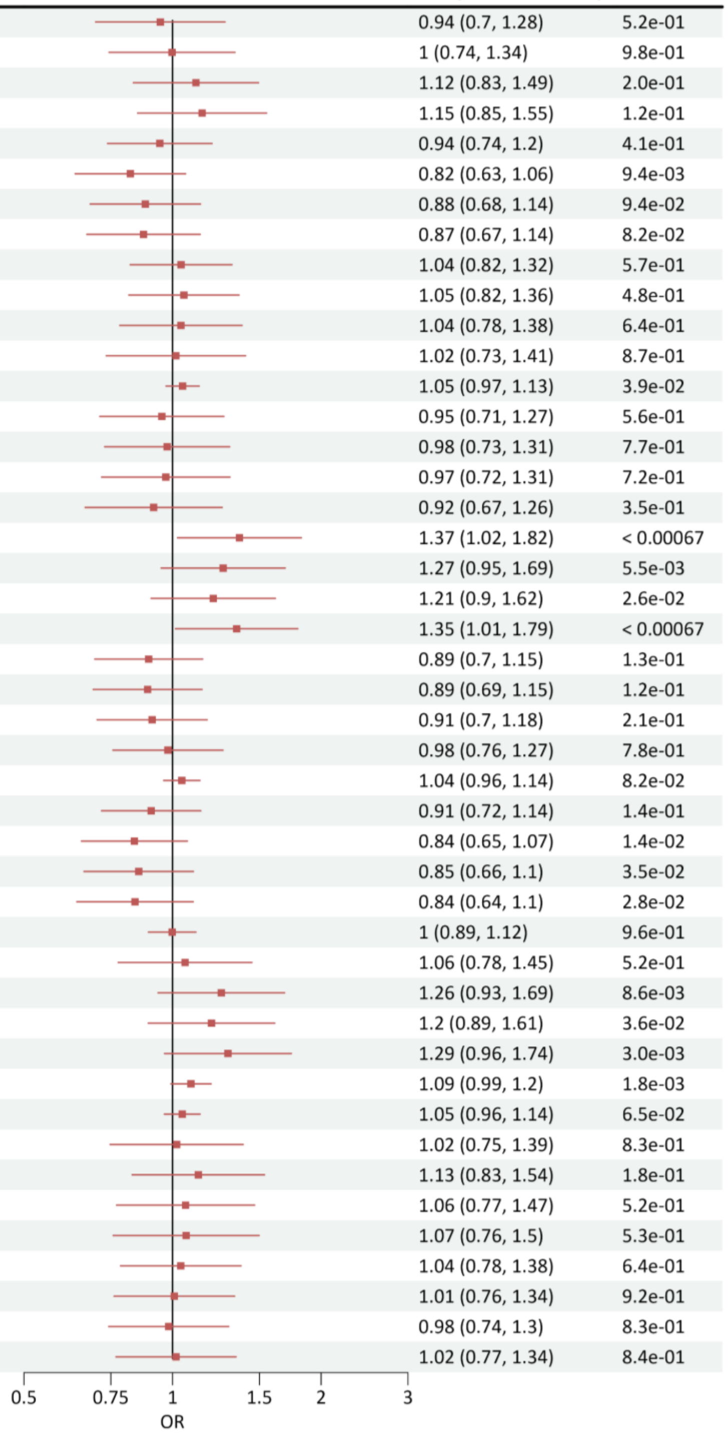

Bonferroni P-Value: 0.00067

C) Comorbidities		OR (Bonferroni CI)	P-Value
Cancer		1.18 (0.99, 1.41)	1.1e-03
Cancer - metastatic		1.54 (1.02, 2.34)	< 0.00067
Cerebrovascular disease		1.09 (0.8, 1.49)	3.5e-01
Congestive heart failure		2.56 (1.85, 3.55)	< 0.00067
Chronic kidney disease		1.36 (1.06, 1.75)	< 0.00067
COPD		1.42 (1.16, 1.73)	< 0.00067
Dementia		1.33 (0.8, 2.19)	5.6e-02
Diabetes		1.43 (1.11, 1.84)	< 0.00067
Fracture		1.09 (0.84, 1.42)	2.6e-01
Hemiplegia		1.34 (0.74, 2.43)	9.9e-02
Liver disease - moderate to severe		1.27 (0.77, 2.07)	1.0e-01
Myocardial infarction		2.33 (1.8, 3.02)	< 0.00067
Peptic ulcer		1.43 (1.16, 1.76)	< 0.00067
Peripheral vascular disease		1.48 (1.03, 2.12)	< 0.00067
Rheumatoid arthritis		1.55 (1.12, 2.14)	< 0.00067

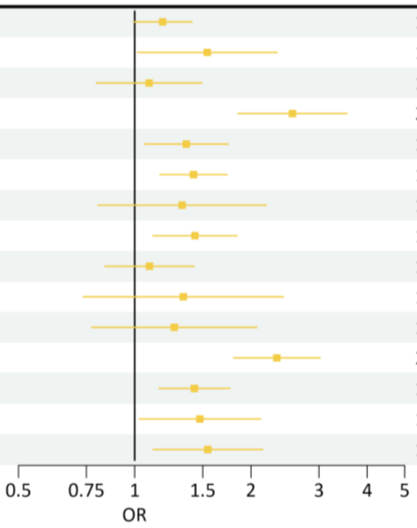

Bonferroni P-Value: 0.00067

Supplementary Figure 11. Long COVID cohort scheme. Participant (1) shows a participant with more than one COVID-19 infection during the study period. Notice that only the latest COVID-19 infection is included. Participant (2) is excluded as it did not get infected by COVID-19 during the study period. Participant (3) is excluded as it was infected by COVID-19 within 30 days before answering the COVID-19 questionnaire.

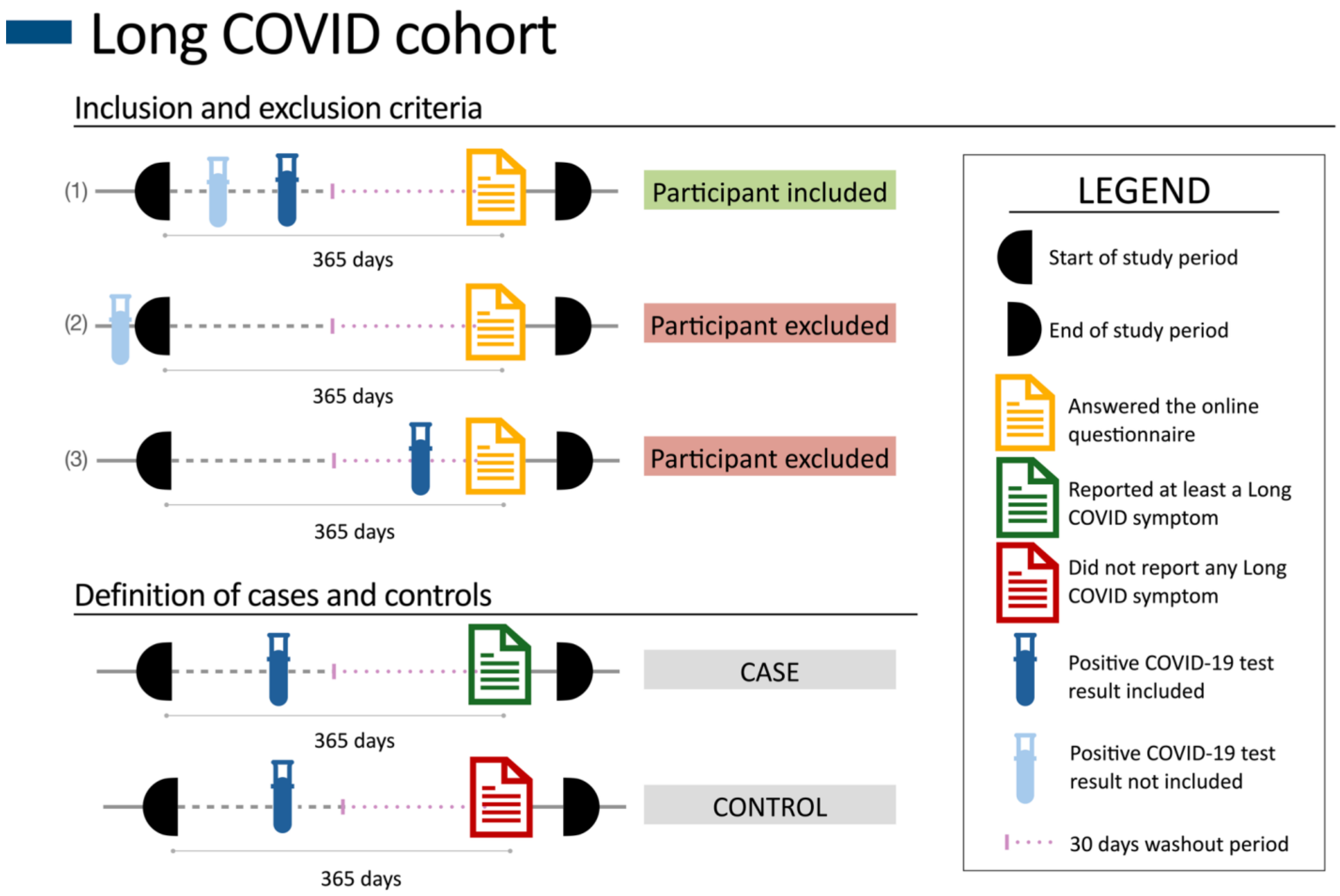

Supplementary Figure 12. PACS cohort scheme. Each grey rectangle represents one participant. Each horizontal line within a rectangle corresponds to a separate COVID-19 infection. *Inclusion and exclusion criteria:* Participant (1) has three infections but is excluded of the cohort as they received a PACS diagnosis within 365 days before their first infection, violating an inclusion criteria. Participant (2) has two infections. While during their first infection they meet the inclusion criteria, the second does not, as they receive a PACS diagnosis within 30 days of infection. Therefore, this participant is excluded. Participant (3) also has two infections, both meeting inclusion criteria. Hence, they are included in the analysis. *Definition of cases and controls:* Participant (4) has two infections. During the second one, they receive a PACS diagnosis between 30 days and 365 days post-infection and are therefore classified as a PACS case. Participant (5) has two infections, neither followed by a PACS diagnosis within the specified window and is classified as a PACS control.

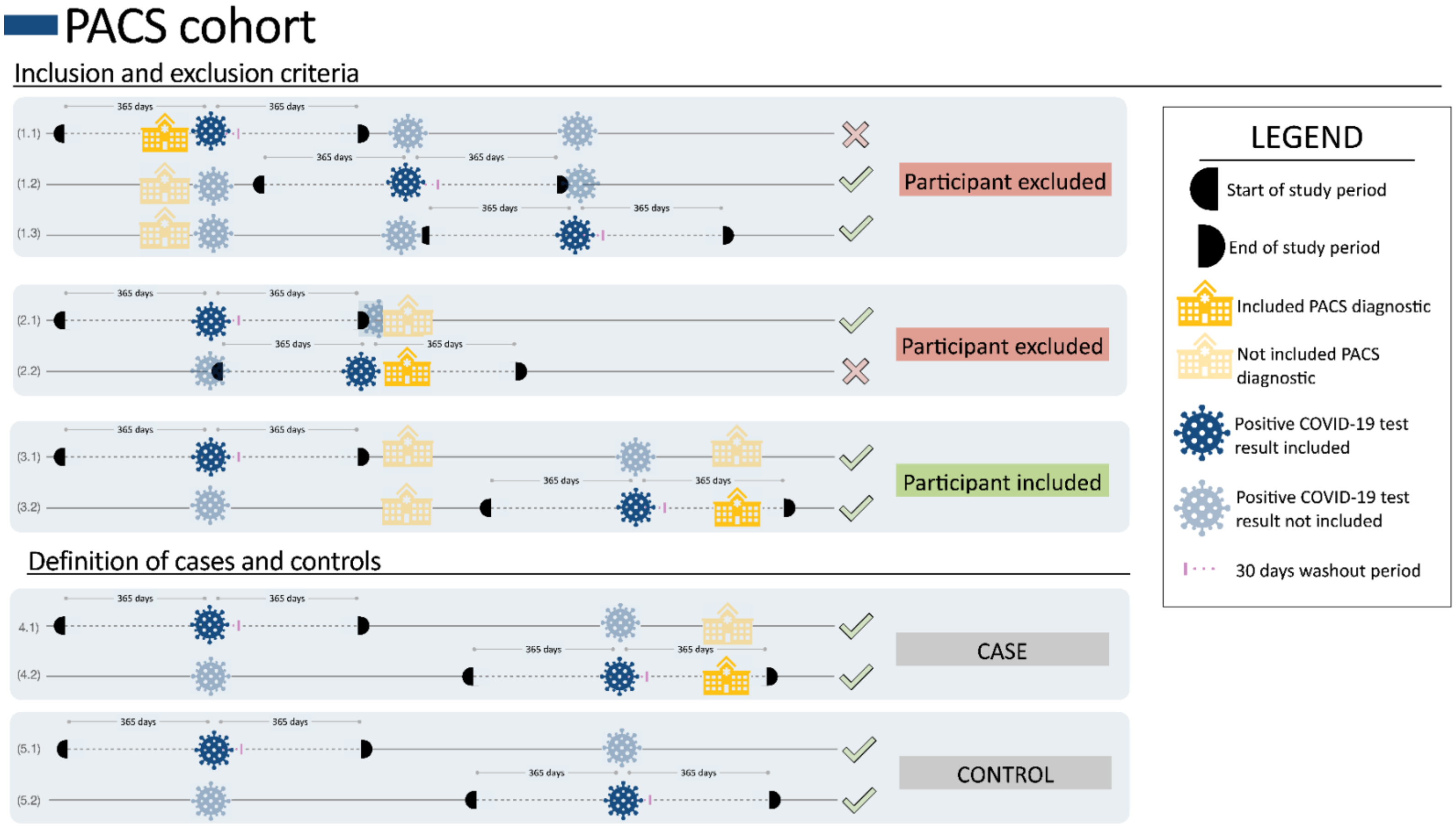

References

1. Sterne JA, White IR, Carlin JB, et al. Multiple imputation for missing data in epidemiological and clinical research: potential and pitfalls. *BMJ.* 2009;338:b2393.
