# Supplementary material for: Sociodemographic factors, biomarkers and comorbidities associated with post-acute COVID-19 sequelae in UK Biobank
Source: Nat Commun. 2025 Jul 30;16:7009. doi: 10.1038/s41467-025-62354-0 (PMC12311030; doi:10.1038/s41467-025-62354-0)
Supplement: Supplementary file 2 — Reporting Summary [file 41467_2025_62354_MOESM2_ESM.pdf]

nature portfolio

Corresponding author(s): Daniel Prieto-Alhambra

Last updated by author(s): 16/07/2025

# Reporting Summary

Nature Portfolio wishes to improve the reproducibility of the work that we publish. This form provides structure for consistency and transparency in reporting. For further information on Nature Portfolio policies, see our Editorial Policies and the Editorial Policy Checklist.

## Statistics

For all statistical analyses, confirm that the following items are present in the figure legend, table legend, main text, or Methods section.

| n/a | Confirmed | |
|---|---|---|
| ☐ | ☒ | The exact sample size ($n$) for each experimental group/condition, given as a discrete number and unit of measurement |
| ☒ | ☐ | A statement on whether measurements were taken from distinct samples or whether the same sample was measured repeatedly |
| ☒ | ☐ | The statistical test(s) used AND whether they are one- or two-sided<br>*Only common tests should be described solely by name; describe more complex techniques in the Methods section.* |
| ☐ | ☒ | A description of all covariates tested |
| ☐ | ☒ | A description of any assumptions or corrections, such as tests of normality and adjustment for multiple comparisons |
| ☐ | ☒ | A full description of the statistical parameters including central tendency (e.g. means) or other basic estimates (e.g. regression coefficient) AND variation (e.g. standard deviation) or associated estimates of uncertainty (e.g. confidence intervals) |
| ☐ | ☒ | For null hypothesis testing, the test statistic (e.g. $F$, $t$, $r$) with confidence intervals, effect sizes, degrees of freedom and $P$ value noted<br>*Give P values as exact values whenever suitable.* |
| ☒ | ☐ | For Bayesian analysis, information on the choice of priors and Markov chain Monte Carlo settings |
| ☒ | ☐ | For hierarchical and complex designs, identification of the appropriate level for tests and full reporting of outcomes |
| ☐ | ☒ | Estimates of effect sizes (e.g. Cohen's $d$, Pearson's $r$), indicating how they were calculated |

*Our web collection on statistics for biologists contains articles on many of the points above.*

## Software and code

Policy information about availability of computer code

| | |
|---|---|
| Data collection | The UK Biobank performed all data collection procedures centrally, and no additional data was specifically obtained for this study. |
| Data analysis | Data manipulation was done with R software (version 4.3.0), and the main packages used for analysis and reporting include mice, dplyr (version 1.1.3), ggplot2 (version 3.5.1), and glmnet (version 4.1.8). Our analytical code is publicly available in the following GitHub repository: https://github.com/oxford-pharmacoepi/Determinants_LC_PACS |

For manuscripts utilizing custom algorithms or software that are central to the research but not yet described in published literature, software must be made available to editors and reviewers. We strongly encourage code deposition in a community repository (e.g. GitHub). See the Nature Portfolio guidelines for submitting code & software for further information.

## Data

Policy information about availability of data

All manuscripts must include a data availability statement. This statement should provide the following information, where applicable:

- Accession codes, unique identifiers, or web links for publicly available datasets
- A description of any restrictions on data availability
- For clinical datasets or third party data, please ensure that the statement adheres to our policy

The UK Biobank patient-level data are available under restricted access for bona fide researchers, access can be obtained by applying at http://ukbiobank.ac.uk/register-apply/. Raw data are protected and are not available due to data privacy laws. All participants provided informed written consent to take part in the study.

Ethics approval for the UK Biobank was granted by the North West Multi-Centre Research Ethics Committee in 2006 and was updated regularly after that (https://www.ukbiobank.ac.uk/learn-more-about-uk-biobank/about-us/ethics). This study was conducted after approval by UK Biobank under application reference 151425.

## Research involving human participants, their data, or biological material

Policy information about studies with human participants or human data. See also policy information about sex, gender (identity/presentation), and sexual orientation and race, ethnicity and racism.

| | |
|---|---|
| Reporting on sex and gender | We incorporated self-reported sex data into our analysis. Self-reported sex served both as a covariate and an exposure. Table 1 of the manuscript reports the percentage of males and females in each cohort. |
| Reporting on race, ethnicity, or other socially relevant groupings | We incorporated ethnicity data into our analysis. Self-reported ethnicity served both as a covariate and an exposure. Table 1 of the manuscript reports the percentage of white and non-white individuals in each cohort. |
| Population characteristics | Table 1 shows the baseline characteristics for each study cohort. |
| Recruitment | The UK Biobank is a prospective cohort study which recruited over 500,000 participants between 2006-2010. The UK Biobank study, as well as the recruitment process, has been described in detail by Bycroft et al., Nature 2018 (https://www.nature.com/articles/s41586-018-0579-z). UK Biobank participants were volunteers. Hence, they are acknowledged to be healthier than the general population |
| Ethics oversight | Ethics approval for the UK Biobank was granted by the North West Multi-Centre Research Ethics Committee in 2006 and was updated regularly after that (https://www.ukbiobank.ac.uk/learn-more-about-uk-biobank/about-us/ethics). All participants provided informed written consent to take part in the study and be followed-up through linkage to health-related records. This study received ethical approval from the UKBB Ethics Advisory Committee (EAC) under application 151425. |

Note that full information on the approval of the study protocol must also be provided in the manuscript.

# Field-specific reporting

Please select the one below that is the best fit for your research. If you are not sure, read the appropriate sections before making your selection.

☒ Life sciences ☐ Behavioural & social sciences ☐ Ecological, evolutionary & environmental sciences

For a reference copy of the document with all sections, see nature.com/documents/nr-reporting-summary-flat.pdf

# Life sciences study design

All studies must disclose on these points even when the disclosure is negative.

| | |
|---|---|
| Sample size | Sample size was not predetermined. The sample size was determined by the maximum number of participants in the UK Biobank that followed the inclusion criteria specified in Supplementary Figure 1. |
| Data exclusions | We did not exclude any data. |
| Replication | We did not have a separate replication cohort. Reproducibility of the analysis should be possible if researchers have access to the UK Biobank dataset, and follow the publicly available analytical code. |
| Randomization | Randomization was not relevant in our study as it is not within the causal inference framework. Instead, we performed a hypothesis free analysis . |
| Blinding | Blinding was not applicable as no intervention were applied to participants |

# Reporting for specific materials, systems and methods

We require information from authors about some types of materials, experimental systems and methods used in many studies. Here, indicate whether each material, system or method listed is relevant to your study. If you are not sure if a list item applies to your research, read the appropriate section before selecting a response.

## Materials & experimental systems

| n/a | Involved in the study |
|---|---|
| ☒ | ☐ Antibodies |
| ☒ | ☐ Eukaryotic cell lines |
| ☒ | ☐ Palaeontology and archaeology |
| ☒ | ☐ Animals and other organisms |
| ☒ | ☐ Clinical data |
| ☒ | ☐ Dual use research of concern |
| ☒ | ☐ Plants |

## Methods

| n/a | Involved in the study |
|---|---|
| ☒ | ☐ ChIP-seq |
| ☒ | ☐ Flow cytometry |
| ☒ | ☐ MRI-based neuroimaging |

# Plants

| | |
|---|---|
| Seed stocks | *Report on the source of all seed stocks or other plant material used. If applicable, state the seed stock centre and catalogue number. If plant specimens were collected from the field, describe the collection location, date and sampling procedures.* |
| Novel plant genotypes | *Describe the methods by which all novel plant genotypes were produced. This includes those generated by transgenic approaches, gene editing, chemical/radiation-based mutagenesis and hybridization. For transgenic lines, describe the transformation method, the number of independent lines analyzed and the generation upon which experiments were performed. For gene-edited lines, describe the editor used, the endogenous sequence targeted for editing, the targeting guide RNA sequence (if applicable) and how the editor was applied.* |
| Authentication | *Describe any authentication procedures for each seed stock used or novel genotype generated. Describe any experiments used to assess the effect of a mutation and, where applicable, how potential secondary effects (e.g. second site T-DNA insertions, mosiacism, off-target gene editing) were examined.* |
